# Measuring people’s covariational reasoning in Bayesian situations

**DOI:** 10.3389/fpsyg.2023.1184370

**Published:** 2023-10-16

**Authors:** Nicole Steib, Stefan Krauss, Karin Binder, Theresa Büchter, Katharina Böcherer-Linder, Andreas Eichler, Markus Vogel

**Affiliations:** ^1^Mathematics Education, Faculty of Mathematics, University of Regensburg, Regensburg, Germany; ^2^Mathematics Education, Institute of Mathematics, Ludwig Maximilian University of Munich, Munich, Germany; ^3^Mathematics Education, Institute of Mathematics, University of Kassel, Kassel, Germany; ^4^Mathematics Education, Institute of Mathematics, University of Freiburg, Freiburg, Germany; ^5^Mathematics Education, Institute of Mathematics, University of Education Heidelberg, Heidelberg, Germany

**Keywords:** covariational reasoning, Bayesian reasoning, double-tree, unit square, natural frequencies

## Abstract

Previous research on Bayesian reasoning has typically investigated people’s ability to assess a posterior probability (i.e., a positive predictive value) based on prior knowledge (i.e., base rate, true-positive rate, and false-positive rate). In this article, we systematically examine the extent to which people understand the effects of changes in the three input probabilities on the positive predictive value, that is, *covariational reasoning*. In this regard, two different operationalizations for measuring covariational reasoning (i.e., by single-choice vs. slider format) are investigated in an empirical study with *N* = 229 university students. In addition, we aim to answer the question wheter a skill in “conventional” Bayesian reasoning is a prerequisite for covariational reasoning.

## 1. Introduction

Imagine a police officer who frequently conducts traffic stops and uses breathalyzer tests in order to determine whether a driver is intoxicated. She has noticed over time that breathalyzer test results are sometimes discovered to have been false positives (ascertained by ensuing blood-alcohol level lab tests). Then imagine that it is early morning on New Year’s Day, and the number of people driving under the influence of alcohol is substantially higher than on an average day. To her surprise, the officer finds that the test results from this time period, when the number of people driving under the influence of alcohol is higher than on average, seem to be more reliable. Therefore, she wonders why the test does not always *work in the same way,* and asks herself if and how a changed amount of intoxicated people might affect the validity of a positive test result.

The calculation of the so-called *positive predictive value* (*PPV*)—that is, in this specific Bayesian situation, the probability of an individual actually being under the influence of alcohol given a positive breathalyzer test result—is usually called *Bayesian reasoning* and has been examined in various studies (Talboy and Schneider, 2017; Reani et al., 2018; Brase, 2021). In general, a “conventional” Bayesian situation consists of a binary hypothesis, for example being under the influence of alcohol *H* or not being under the influence of alcohol $\bar{H}$, and a binary indicator, that is, a positive test result *I* or a negative test result $\bar{I}$ (Zhu and Gigerenzer, 2006). Experimental cognitive psychology thus far has focused almost exclusively on the computation of a correct answer in Bayesian tasks when three pieces of information are given, namely base rate, true-positive rate, and false-positive rate. In Table 1, we refer to this conventional task as “calculation.”

**Table 1:** Calculation and covariation tasks.

*Extension of the concept Bayesian reasoning*				
	Formal notation	Technical term	Calculation *Conventional Bayesian reasoning task:*	Covariation *Possible instructions regarding covariation:*
Given information	P(*H*)	Base rate (*b*)	The probability is 10% that a person who undergoes a breathalyzer test is under the influence of alcohol.	Imagine the probability that a person is actually under the influence of alcohol is 2% smaller than 10%. (concrete change)
	P(*I*|*H*)	True-positive rate (*t*)	If a person is actually under the influence of alcohol, the probability is 90% that this person will test positive.	Imagine that the probability of a person under the influence of alcohol actually testing positive is smaller than 90%. (qualitative change)
	P(*I*|$\bar{H}$)	False-positive rate (*f*)	If a person is not under the influence of alcohol, the probability is 50% that this person will nevertheless test positive.	Imagine that the probability of a person not under the influence of alcohol falsely testing positive is actually 3% smaller than 50%. (concrete change)
Question	P(*H*|*I*)	Positive predictive value (*PPV*)	If a person tests positive, what is the probability that this person is under the influence of alcohol?	How does that change the probability that a person is actually under the influence of alcohol, if he or she tests positive?

Even though Bayesian situations can be extremely relevant in various domains, and a misinterpretation can have serious consequences (Hoffrage et al., 2000; Spiegelhalter et al., 2011), calculation tasks usually cannot be solved by participants (McDowell and Jacobs, 2017), nor by students (Binder et al., 2015), nor by experts, for example from the fields of medicine (Hoffrage and Gigerenzer, 1998; Garcia-Retamero and Hoffrage, 2013) or law (Hoffrage et al., 2000; Lindsey et al., 2003). Difficulties with conventional Bayesian reasoning as well as helpful strategies for calculating the positive predictive value are discussed in section 2.1.

In Bayesian situations with two binary variables, people can easily be confronted with changed input parameters and their influence on e.g., the *PPV* (that we call *covariation* in the following see Table 1): in the case of our introductory example, the fact that it is early morning on New Year’s Day means an increase in the base rate that ultimately results in an increase of the *PPV* (for a theoretical analysis of the respective effects of changes of the three parameters on the *PPV*, see section 2.2). Other examples of relevant changes in the base rate include medical situations. For instance, with COVID-19 tests, it makes a huge difference whether a tested person comes from a high- or a low-incidence area. But also understanding the effect on the *PPV* of changes in true-positive and false-positive rates is of everyday importance, for instance, if a new COVID-19 test becomes available or if the difference between the meaning of a positive rapid test or PCR test has to be understood. Yet there are almost no empirical investigations on people’s understanding of the effects induced by changes in these input probabilities (see, e.g., Borovcnik, 2012). Interestingly, the question of the operationalization of a covariation task is not as straightforward, as we will see in section 2.3.

In the present article, we propose an explicit extension of the research referring to Bayesian reasoning by adding both the aspect of covariation and the corresponding skill of covariational reasoning, as well as how to approach measuring covariational reasoning. Before addressing covariation (2.2) and possible operationalizations to measure people’s respective skills (2.3) theoretically, we first summarize findings and helpful strategies concerning conventional Bayesian reasoning that might also be suitable for covariational reasoning (2.1).

## 2. Theoretical background

### 2.1. Calculation of the positive predictive value as one aspect of Bayesian reasoning

In the following, we call the typically examined conventional Bayesian reasoning (i.e., estimating the *PPV* at a given base rate, true-positive rate, and false-positive rate) *calculation*. In general, the positive predictive value can be assessed (e.g., with Bayes formula):

P(H|I)=P(I|H)·P(H)P(I|H)·P(H)+P(I|H¯)·P(H¯)

In the introductory example, with hypothesis *H* (being under the influence of alcohol) and given statistical information *I* (positive result in the breathalyzer test), the positive predictive value can be calculated as follows (for the specific numbers, see Table 1):

$P(H|I)\;=\;\frac{0.9\;\;\cdot \;\;0.1}{0.9\;\;\cdot \;\;0.1\;\;+0.5\;\;\cdot \;\;0.9}$ ≈ 0.167 = 16.7%.

Many studies have shown that a majority of people fail when solving tasks of this structure (Hoffrage and Gigerenzer, 1998; Garcia-Retamero and Hoffrage, 2013; Binder et al., 2015). A meta-analysis reveals that only 4% of participants are able to make correct inferences (McDowell and Jacobs, 2017).However, research over the past 30 years has shown that there are at least two helpful strategies for solving such tasks: (a) translating the given numerical information from probabilities into what is known as natural frequencies, (e.g., replacing probabilities or percentages like “80% of the people who are under the influence of alcohol test positive” with expressions like “80 out of 100 people who are under the influence of alcohol test positive”; Gigerenzer and Hoffrage, 1995; Krauss et al., 2020), and (b) visualizing the given information (Binder et al., 2015; Böcherer-Linder and Eichler, 2019).

- Natural frequencies

When all statistical information is expressed in terms of *natural frequencies* instead of probabilities, one can imagine actual people, and the solution algorithm becomes simpler (Gigerenzer and Hoffrage, 1995; McDowell and Jacobs, 2017). In the format of natural frequencies, the above-mentioned Bayesian situation about breathalyzer tests (Table 1) translates to the following:

*Given information*:

100 out of 1,000 people (who participate in breathalyzer tests) are under the influence of alcohol. 90 out of these 100 people who are under the influence of alcohol test positive. 450 out of the 900 people who are not actually under the influence of alcohol nevertheless test positive.

*Question:*

How many of the people who test positive in the breathalyzer test are under the influence of alcohol?

Now, one can see that 90 + 450 people test positive with the breathalyzer test, and that 90 out of these 540 people who have tested positive are actually under the influence of alcohol. The above-mentioned meta-analysis found that study participants’ average performance increases up to 24% in natural frequency versions (McDowell and Jacobs, 2017).

- Visualizations

**Figure 1:**
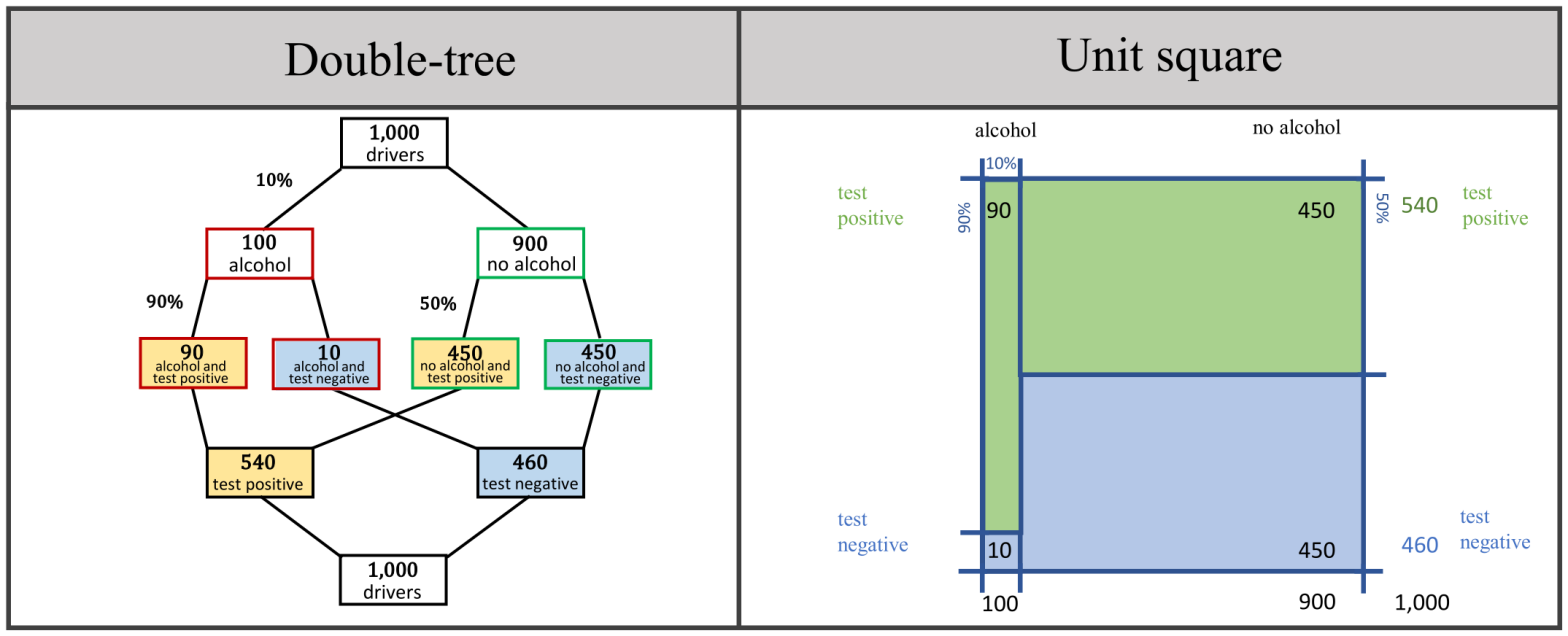
Double-tree and unit square as visualizations in the Bayesian situation about the breathalyzer test. Note that neither natural frequencies nor visualizations are a factor of interest in the present study. Both tools will be used experimentally to provide an understanding of conventional Bayesian reasoning situations and thus to make it possible to investigate covariational reasoning at all.

*Visualizations* can also be a helpful tool for improving conventional Bayesian reasoning (e.g., Brase, 2014; Sirota et al., 2014). Typical visualizations are, for example, tree diagrams or 2 × 2 tables (for an overview of a variety of alternative visualizations such as roulette-wheel diagrams or frequency grids, see Spiegelhalter et al., 2011 or Binder et al., 2015). In the present study, we chose enhancements of tree diagrams and 2 × 2 tables, namely double-trees (Binder et al., 2022) and unit squares (Böcherer-Linder and Eichler, 2017; Pfannkuch and Budgett, 2017; Talboy and Schneider, 2017). Both visualizations are suited to calculation and covariation as well, and can, in principle, be equipped with probabilities and/or absolute frequencies. In Figure 1 both visualizations display the Bayesian situation about the breathalyzer test.

### 2.2. Covariation as an extension of Bayesian reasoning

In mathematics, functions can display the *covariation* between two variables *x* and *y* (e.g., *y*(*x*) = *x*^2^). The concept of covariation—which is prominent in the field of mathematics education—stresses the mutual, dynamic association between the independent variable *x* and the dependent variable *y*(*x*). Mathematics educators who are interested in *functional thinking* empirically inverstigate, for instance, students’ understanding of the dynamic relation between (changes of) *x* and (changes of) *y*(*x*) (e.g., Thompson and Harel, 2021). For example, for the function *y*(*x*) = *x*^2^, there is a *quadratic relation* between *x* and *y*, meaning, for instance, that doubling the *x*-value results in quadrupling the *y*-value.

From the perspective of mathematics education, “covariation” is one of three “basic ideas” (“Grundvorstellungen”) of proper functional thinking (Vollrath, 1989). The other two are “mapping” (the *x*-value of 2 *is assigned to* the *y*-value of 4) and “function as an object” (e.g., in this case, the object represented by a parabolic graph).

From a mathematical point of view, Bayes’ theorem cannot only be understood as a *formula* but also as a *function* that expresses the dependency of the positive predictive value (*PPV*) on three variables, namely the base rate (*b*), the true-positive rate (*t*), and the false-positive rate (*f*):


(1) $$PPV\left(b,\;t,\;f\right)=\frac{t\;\cdot \;b}{t\;\cdot \;b+f\;\cdot \;\left(1-b\right)}$$

**Figure 2:**
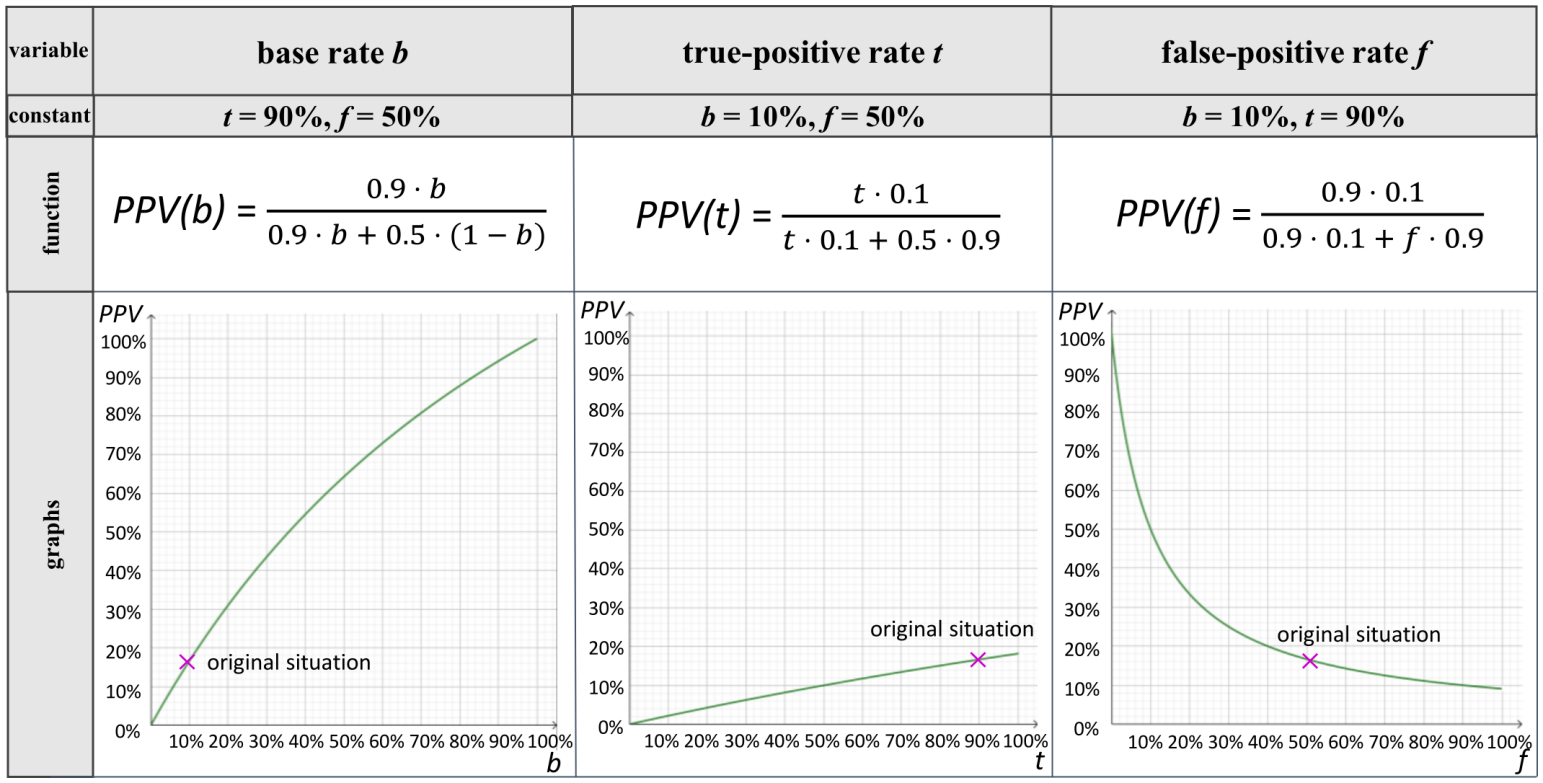
Graphs for the *PPV* as function in the Bayesian situation about the breathalyzer test when the base rate (*b*) (column 1), the true-positive rate (*t*) (column 2), and the false-positive rate (*f*) (column 3) change individually.

If in equation (1), two of the three parameters are fixed as constant and one is considered “variable,” one gets the three functions *PPV(b)*, *PPV(t)*, and *PPV(f)* (the functions are plotted in Figure 2). A typical question considering covariation in the field of Bayesian reasoning might be: “How does the positive predictive value *change* when the base rate (considered as a variable) increases/decreases (and the other two parameters remain unchanged)?” In general, in this article, we use the term *covariational reasoning* for participants’ understanding of the effects when *one* of the three variables (*b*, *t*, *f*) changes.

Covariation between the *PPV* and each of the three variables can be illustrated using graphs (Figure 2; graphs are not experimentally implemented in the present approach).

**Figure 3:**
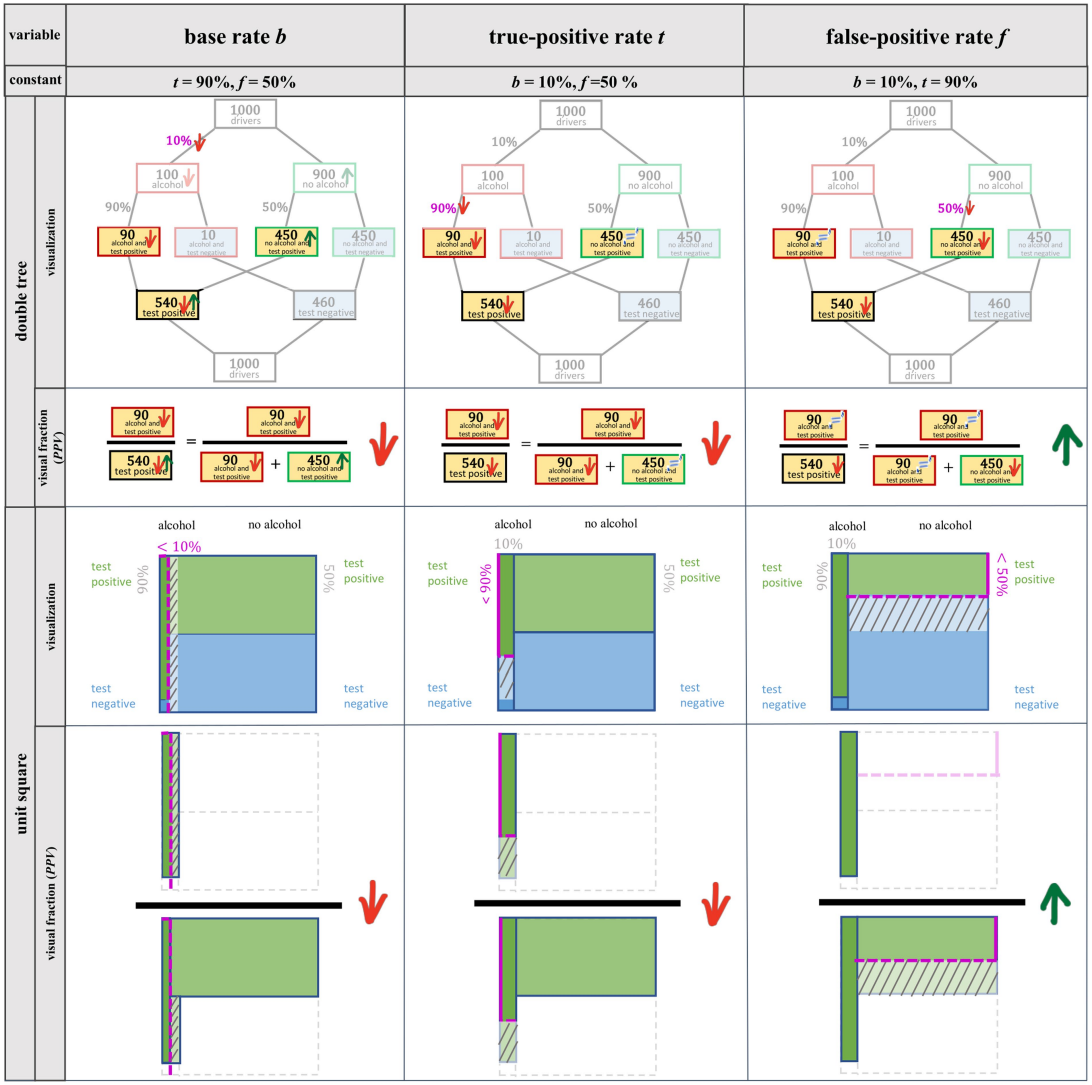
Double-tree diagram with visual fraction and unit square with visual fraction for the reduction of *b* (column 1), *t* (column 2), and *f* (column 3).

Alternatively, the idea of covariation can be illustrated by means of a double-tree diagram and a unit square (Figure 1). Figure 3 depicts decreases of *b*, *t*, and *f* in the Bayesian situation about the breathalyzer test. In the double-tree diagram, arrows indicate which of the frequencies (or parameters) change and in which direction. In the unit square, the shifted lines indicate the changes. In the line below each visualization, the effects on the *PPV* are shown using a *visual fraction* (Eichler and Vogel, 2010; Büchter et al., 2022a).

In the following, we describe in detail the effects of changes in *b*, *t*, and *f* on the *PPV* by means of a double-tree diagram and unit square (for a summary, see Table 2). At the end of each subsection (2.2.1–2.2.3), the range of possible changes as displayed by graphs (Figure 2) is discussed.

**Table 2:** Resulting changes of the PPV (right column) dependent on the changes of input variables (left column); in both middle columns already the indeed in the empirical study implemented changes are displayed (see section 4. Empirical study).

Given parameter change	Implemented in …		Resulting change of *PPV*
	Single-choice	Slider	
Base rate (*b*) 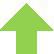 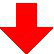	—	*2%*	*PPV* 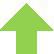
	*Qualitative*	—	*PPV* 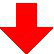
*True-positive rate (t)* 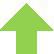 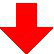	*Qualitative*	—	*PPV* 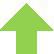
	—	*3%*	*PPV* 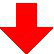
*False-positive rate (f)* 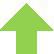 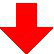	*Qualitative*	—	*PPV* 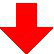
	—	*3%*	*PPV* 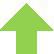

#### 2.2.1. Changing the base rate (*b*)

Considering a possible decrease in *b* in the context of the breathalyzer test (Figure 3, column 1) means that the probability of a person being under the influence of alcohol is smaller than 10%. The frequency (in the double-tree) or area (in the unit square) of persons who are under the influence of alcohol now becomes smaller than in the original situation, and thus the relevant quantity of *true-positives*—the number of people who are under the influence of alcohol and receive a positive test result—also becomes smaller. As a consequence, the frequency/area of persons who are not under the influence of alcohol increases, and thus the relevant quantity of *false-positives*—the number of people who are not under the influence of alcohol yet receive a positive test result—increases as well. In both corresponding visual fractions that represent the *PPV* (column 1), the numerator (e.g., true-positives) decreases, and in the denominator, the first summand (e.g., true-positives) decreases while the second summand (e.g., false-positives) increases. However, it is unclear at first sight in the visual fraction regarding the double-tree whether the denominator increases or decreases. In any case, the denominator cannot decrease as much (relatively and absolutely) as the numerator. Therefore, the fraction (and thus the positive predictive value) *decreases* with a *decrease* in the base rate. If the base rate were to *increase*, all frequencies would change in the opposite of direction and the *PPV* would *increase* as well. Thus from a mathematical point of view, increase and decrease in statistical information work analogously (see Table 2). Note that only in this case (2.2.1) do all relevant frequencies change in numerator and denominator as well which is why this change is considered the most difficult.

In Figure 2 (column 1), it can be observed that a small change in *b* has quite a large influence on the change in *PPV* in the given context. This is even more the case for small base rates when the false-positive rate is also low (e.g., 5%). Moreover, the *PPV* can take any value from 0% to 100% when the base rate changes. When the other two parameters are varied (2.2.2 and 2.2.3), it is typically not the case that the *PPV* can take any value between 0% and 100% (see Figure 2, columns 2–3).

#### 2.2.2. Changing the true-positive rate (*t*)

A decrease in *t* in the context of the breathalyzer test (Figure 3, column 2) means that the probability of receiving a positive test result when a person is under the influence of alcohol is smaller than 90%. This reduces the relevant frequency/area of the true-positives. Since the frequency/area of the people who are not under the influence of alcohol remains unchanged, the relevant false-positives stay the same as well. For the visual fractions (column 2), the numerator becomes smaller and the denominator, in absolute terms, decreases by the same amount, so that the fraction corresponding to the *PPV* becomes smaller. Analogously, *increasing* the true-positive rate would *increase* the *PPV*, since everything would behave exactly the opposite. Contrary to a base-rate change, changes in *t* only result in a change in true-positives (both in the numerator and the denominator of the visual fraction), not in changes in false-positives (in the denominator).

Looking at Figure 2 (column 2), it becomes clear that changes in *t* in the context of the breathalyzer test have a smaller effect on the *PPV* than changes in *b*. With the maximal change of 100% in *t* in the given context, the *PPV* only changes by 20% in total.

#### 2.2.3. Changing the false-positive rate (*f*)

A reduction in *f* in the context of the breathalyzer test (i.e., the probability of receiving a positive result from the test even though one is not under the influence of alcohol) is illustrated in Figure 3 (column 3). The decrease in *f* reduces the frequency/area of false-positives. The frequency/area of true-positives, however, does not change. In the visual fractions (column 3), the numerator as well as the first summand in the denominator (i.e., true-positives) remain the same, and the second summand in the denominator (i.e., false-positives) decreases, so that the fraction corresponding to the *PPV* increases. In the same way, *increasing f* would *decrease* the *PPV*.

In terms of the graphs shown in Figure 2 (column 3), it is clear that in that specific case, small changes in *f* have a large effect on the *PPV* especially for very small false-positive rates. For example, with a false-positive rate of 0%, the positive predictive value would be 100%, while with a false-positive rate of 10%, the positive predictive value already decreases to 50%. The larger *f*  becomes, the smaller the impact on the *PPV* becomes. This strong influence is due to the small given base rate, which is, however, typical for many Bayesian situations (especially in medical contexts, where the base rate denotes the prevalence of diseases).

### 2.3. Measuring covariational reasoning

Interestingly, it is no straightforward task to measure the covariational reasoning of participants. In contrast to Bayesian calculation tasks—where one can just ask for a certain conditional probability (given three other probabilities)—how to operationalize such a task is an open question. Why is measuring covariational reasoning so difficult? If you just change one of the given probabilities (e.g., by a certain percentage), you get nothing more than a new conventional Bayesian reasoning task. For this purpose, we used the single-choice operationalization presented by Böcherer-Linder et al. (2017) for measuring covariational reasoning in Bayesian situations and added a second operationalization with a slider.

#### 2.3.1. Single-choice operationalization

The change in a given probability could be described purely qualitatively without specifying the concrete values of a change (e.g., Table 1 change in *t*). For instance, participants may be guided to imagine that—with reference to a typical Bayesian task—“one of the parameters is now smaller/larger” than the original value. Afterwards, for instance, a closed-ended question format might be implemented asking for the respective effect on the *PPV* (e.g., “increases”, “stays the same”, or “decreases”; see section 4.2).

**Figure 4:**
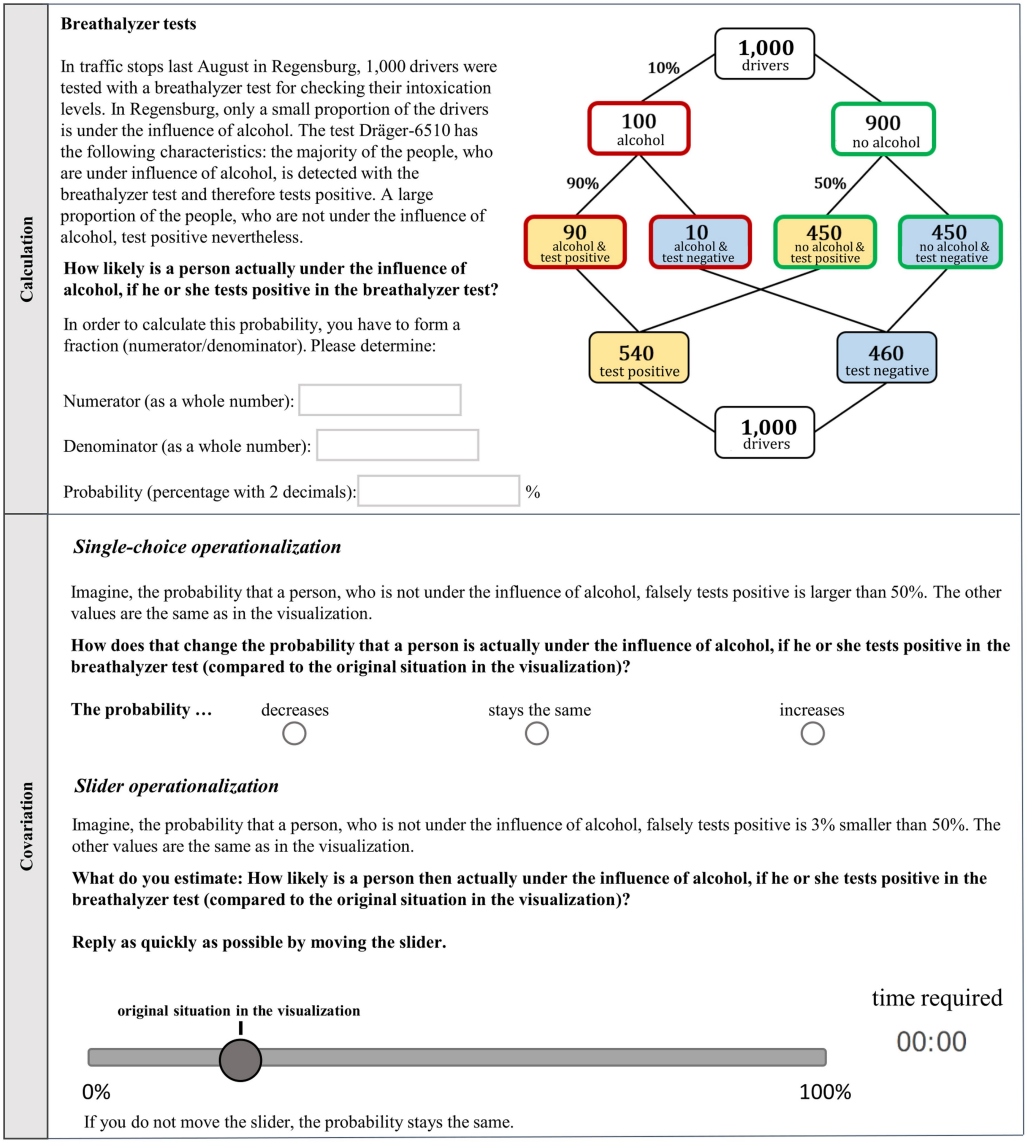
Examples of a calculation task (above) and covariation instructions (below) for a change in *f* (increase in single-choice operationalization and decrease in the slider operationalization).

#### 2.3.2. Slider operationalization

Alternatively, covariational reasoning might be measured with the help of a slider. A concrete change of a parameter could be described (e.g., “the base rate is 2% smaller than 10%”) and a new calculation of the changed *PPV* could be avoided, for example, by introducing time pressure. For instance, a slider for the *PPV* with values from 0% to 100% might be implemented (see section 4.2), and participants could be asked to move the slider—as quickly as possible—from the original position (which resembles the *PPV* in the original Bayesian situation) to the new position, all while a timer is running. In principle, the slider format would allow one to evaluate not only the direction of change but also the participants’ estimations of the degree of this change.

#### 2.3.3. Other possible operationalizations

Other possibilities for measuring covariational reasoning would be, for example, to (openly) ask for concrete reasons for changes in the *PPV*. In this way, the thought processes involved in solving covariation tasks might be recorded. Of course, a combination of single-choice and/or slider with the analysis of possible reasons would also be conceivable for such tasks.

### 2.4. The distinction between the terms “covariation assessment” and “covariational reasoning”

It is important to note that the idea of *covariational reasoning* is different from the concept of *covariation assessment*, which has been used by McKenzie (1994, 2004), McKenzie and Mikkelsen (2007), and Shaklee and Mims (1981). According to McKenzie (1994), covariation assessment refers to the detection of whether two binary variables (e.g., hypothesis *H* and indicator *I*)—e.g., given as numbers in a 2 × 2 table—covary at all. For example, the four frequencies of joint events might be given and the participants have to indicate the strength of contingency on a 100-point scale from 0 (no relation between the two variables) to 100 (a “total relation” between the two variables Arkes and Harkness, 1983). In a similar kind of task created by Shaklee and Mims (1981), the four frequencies of joint events were also given, and then the participants are asked to compare P(I|H) and $P\left(I|\bar{H}\right)$, that is, whether P(I|H) > $P\left(I|\bar{H}\right)$, P(I|H) = $P\left(I|\bar{H}\right),\;or\;P\left(I|H\right)$ < $P\left(I|\bar{H}\right)$ holds.

Note that in contrast, in a typical Bayesian situation, this covariation *is given implicitly* in its framing as a *diagnostic situation*. This means that concepts such as true-positive rate P(I|H)and false-positive rate$\;P\left(I|\bar{H}\right)$ only make sense if $P\left(I|H\right)\neq P\left(I|\bar{H}\right)$. If both of these probabilities were equal, the probability of getting a positive test result would—paradoxically—be independent of health status (and so the medical test would “have nothing to do” with the disease). Therefore, the *given inequality of both conditional probabilities* already states their mutual dependence as understood in the covariation assessment. Consequently, the latter paradigm is not focused on within this article in the context of Bayesian reasoning.

## 3. Research questions

The core questions of the present study are whether people are able to correctly estimate the effects of changes in the given probabilities (*b*, *t*, and *f*) on the *PPV* (“covariational skill”) and how these skills depend on the chosen measurement operationalization (i.e., single-choice vs. slider). Furthermore, we are interested in interactions in this regard concerning the type of the varying input variable, namely base-rate (*b*), true-positive-rate (*t*), and false-positive-rate (*f*).

Another interesting research question for us was the extent to which an ability in conventional Bayesian reasoning (“calculation”) is helpful or is even a prerequisite for successfully applying covariational reasoning. Because of this interest, we implemented a conventional Bayesian reasoning task that was given to participants before the covariation tasks. In order to avoid floor effects (remember the typical performance of 4% when given a probability version, c.f. McDowell and Jacobs, 2017), we made use of visualizations that are well suited for calculation and covariation. Considering the multiple representations of covariation in 2.2, the question arises which visualization should be implemented in order to allow a understanding of the previous conventional Bayesian reasoning task. Since both the formulas and the graphical representation (Figure 2) are based on probabilities (which has proven to be a disadvantageous format in many studies), we chose double-tree diagrams and unit squares (which have already proven helpful in conventional Bayesian reasoning; Böcherer-Linder and Eichler, 2017; Binder et al., 2022). The structure of the visualization was explained to participants in advance in written form using a different context (Supplementary material 1S, 2S). For cross-validation, we implemented two contexts (breathalyzer and mammography). Instead of implementing the context of the COVID-19 test (given above), we chose the well-known mammography task as a medical example in order to be able to compare performances with previous research.

In sum, research question 1 (RQ 1; manipulation check) investigates whether context or visualization type affects calculation in the primary Bayesian task (if this is *not* the case, we can examine covariational skills aggregated across context and visualization). In research question 2 (RQ 2) we address covariational skills.

### 3.1. RQ 1 calculation

1.1Is conventional Bayesian reasoning affected by context (breathalyzer vs. mammography)?

1.2.Is conventional Bayesian reasoning affected by visualization type (double-tree vs. unit square)?

Since RQ 1 focuses on conventional Bayesian reasoning—without referring to covariation—we will be able to compare these results with previous studies.

### 3.2. RQ 2 covariation

2.1 Can people judge the effect of parameter changes on the *PPV* (at all)? Are there differences regarding the type of changed input variable, that is, when considering

- base rate changes?
- true-positive rate changes?
- false-positive rate changes?

2.2 Are there differences in covariational reasoning with respect to the two measurement operationalizations implemented (single-choice vs. slider)?

2.3 Do covariational reasoning skills depend on the participants’ performance in the previous Bayesian calculation?

## 4. Empirical study

### 4.1. Design

An overview of the design is given in Table 3. Each participant worked on two Bayesian situations (breathalyzer test and mammography screening). For each situation, the participants first had to (a) calculate the positive predictive value (calculation task; see Figure 4, above); the following three tasks were to determine how an increase or decrease of the (b) base rate, (c) true-positive rate, or (d) false-positive rate would affect the *PPV* (covariation tasks; see Figure 4, below). Accordingly, each participant had to work on eight tasks (1a-d, 2a-d). The statistical information (*b*, *t*, *f*) in tasks a-d was given as probabilities in a visualization (double-tree or unit square) that was additionally filled with frequencies (Figure 1). Note that we did not experimentally implement the detailed specifications and elaborations in Figure 3. Rather, participants could apply exactly this kind of reasoning in order to demonstrate their skill in covariational reasoning. For each participant, the visualization, which was not a factor of interest in the present study, was held constant in all tasks (see right column in Table 3).

**Table 3:** Overview of the study design.

	Context covariation tasks 1b-d (after calculation task 1a); covariation tasks 2b-d (after calculation task 2a)				Visualization (no factor of interest)
	Mammography		Breathalyzer		
	Slider	Single-choice	Slider	Single-choice	
*N* = 59	**1**			**2**	Double-tree: *N* = 30
					Unit square: *N* = 29
*N* = 57		**1**	**2**		Double-tree: *N* = 27
					Unit square: *N* = 30
*N* = 57		**2**	**1**		Double-tree: *N* = 30
					Unit square: *N* = 27
*N* = 56	**2**			**1**	Double-tree: *N* = 27
					Unit square: *N* = 29

“**1**” and “**2**” refer to the order of contexts processed.

In this table, for instance, *N* = 56 participants (last line) first had to solve a calculation task (context **1**: breathalyzer), and then had to work on the three corresponding covariation tasks in the single-choice operationalization. After answering a further calculation task (in the other context **2**: mammography), they were provided with the corresponding covariation tasks in the slider operationalization. Out of the *N* = 56 participants, *N* = 27 received a double-tree and *N* = 29 a unit square as visualization throughout all eight tasks.

In the covariation tasks, it was always made clear that in each task, only the change in one input variable of the original Bayesian situation should be considered (see Supplementary material 3S). In one of the two contexts, covariation answers had to be given using a single-choice operationalization with three options: the *PPV* (i) decreases, (ii) stays the same, or (iii) increases. In order to avoid simply having a new calculation task, we did not use concrete probability changes here. Instead, the changes (decreases for *b* and increases for *t* and *f*) were not quantified.

In the other context, participants had to move a slider. The original position of the slider was the correct *PPV* in the previous calculation task (rounded to the nearest whole percent), and the slider could be used to change the *PPV* in intervals of 1% (only the numbers for 0% and 100% were depicted on the scale). A timer was used to impose a time pressure. The participants had to answer the questions as quickly as possible. In the slider format, an increase of 3% in *b*, a decrease of 2% in *t*, and a decrease of 2% in *f* was in both contexts given.

The order of the contexts and the order of the three covariation tasks were varied systematically (Table 3). Participants were allowed to use a calculator when completing the tasks.

### 4.2. Instrument

The conventional Bayesian reasoning tasks (1a and 2a) are formulated as a conditional probability question (Figure 4, above; bold). The base rate (*b*), true-positive rate (*t*), and false-positive rate (*f* ) are not given as textual descriptions as in typical Bayesian reasoning tasks, but are depicted at the respective branches in the double-tree, which additionally is completely filled with absolute frequencies.

In Figure 4, the instructions for a task on covariation for an increase in *f* (single-choice) and a decrease in *f* (slider) are described (the actual changes of *b*, *t*, and *f* as realized in our materials can be seen in the gray-shaded area of Table 2. All tasks−included the tasks based on a unit square−are provided in detail in the Supplementary material 3S).

### 4.3. Participants

*N* = 229 students (*N* = 180 female, *N* = 47 male, and *N* = 2 without indication) who were studying to become primary or secondary school mathematics teachers (*N* = 153 for primary, *N* = 78 for secondary) participated in the present study. The participants were students in Germany at the University of Regensburg (*N* = 114) and the University of Kassel (*N* = 115). They were mostly at the beginning of their studies, with *N* = 189 students in the first to third semester and *N* = 40 students in the fourth or a higher semester (*M* = 1.7; *SD* = 2.3). The participants had not received any prior training in probability. The study was carried out in accordance with the University Research Ethics Standards and written informed consent was obtained. The students were informed that their participation was voluntary and that anonymity was guaranteed.

### 4.4. Coding

#### 4.4.1. Calculation tasks

The correct solution in the context of the breathalyzer test was 16.6% and in the context of the mammography screening 33.8%. An answer was coded as correct if the probability or the fraction (which means *both* numerator and denominator values; i.e., 90/540 or 48/142) was provided correctly (it was sufficient if *either* the correct probability *or* the correct fraction was given). Probability answers were also coded as correct if the solution was rounded up or down to the next full percentage point. For instance, in the context of the breathalyzer test, the correct solution is 16.6%, and therefore answers between 16% and 17% were classified as correct solutions.

#### 4.4.2. Covariation tasks

The correct directions of PPV changes, which depend on the directions of changes in *b*, *t*, and *f*, are depicted on the right in Table 2. In the single-choice operationalization, answers were coded as correct if the right qualitative option for the *PPV* (out of “decreases,” “stays the same,” or “increases”) was chosen. In the slider operationalization, the original slider position was that of the (correct) *PPV* in the previous calculation task (17% or 34%; without the numerical specification of the correct value depicted at the scale, Figure 4). When the slider was moved in the correct direction, the answer was scored as correct. Since the metric *PPV* (0–100%) was divided into three categories by the slider position (“decreases,” “stays the same,” or “increases”), we could also theoretically compare judgments with both operationalizations for measuring covariational reasoning.

### 4.5. Statistical analyses

For each research question, we ran generalized linear mixed models with a logistic link function in order to predict the probability that participants solve a task correctly (as a binary dependent variable, 0 = wrong, 1 = correct).

In terms of RQ 1 (calculation skills), in order to compare the effects of contexts and visualization type, the context “breathalyzer” and the visualization “double-tree” were specified as reference categories. The context “mammography” and the visualization “unit square” were included as explanatory factors via dummy coding. In addition, an interaction term *context × visualization* was modeled. The predicted probability ${\hat{\gamma }}_{ij}$ of solving a calculation task correctly is given by:


$$\begin{array}{c} \;{\hat{\gamma }}_{ij}=\beta_{0}+\beta_{1}\;.\;context_{i}+\beta_{2}\;.\;visualization_{j}\\ +\beta_{3}\;.\;context_{i}\times visualization_{j}\;\;\;\;\left(\mathrm{model}\;\;1\right) \end{array}$$

In terms of RQ 2.1, which relates to covariational reasoning, the factor “type of covariation task” was considered by taking a change of the base rate (*b*) as the reference category and including a change of the true-positive rate (*t*) as well as of the false-positive rate (*f*) as the explanatory factor via dummy coding (change in *t* with 0 and 1; change in *f* with 0 and − 1). The predicted probability ${\hat{\gamma }}_{ij}$ of solving a covariation task correctly is given by:


$${\hat{\gamma }}_{ij}=\beta_{0}+\beta_{1}\;.\;change\_t_{i}+\beta_{2}\;.\;change\_f_{j}\left(\mathrm{model}\;2.1\right)$$

In order to statistically compare the effects of the operationalization for measuring covariational reasoning regarding RQ 2.2 for each type of covariation task (i.e., for changes of *b*, *t*, and *f* separately), the single-choice operationalization was specified as the reference category (with the slider operationalization being the explanatory factor). The predicted probability ${\hat{\gamma }}_{i}$ of solving a covariation task correctly is given by:


$$\;{\hat{\gamma }}_{i}=\beta_{0}+\beta_{1\;}.\;operationalization_{i}\;\left(\mathrm{model}\;2.2\right)$$

Concerning RQ 2.3, we ran a model in which we specified the single-choice operationalization as the reference category $\left(\beta_{1}\right)$ for measuring covariational reasoning and included the slider operationalization (as the explanatory factor) via dummy coding. Furthermore, in this model, the participants who could not previously calculate the positive predictive value (see 5.1) were implemented as another reference category ($\beta_{2}),$ and the factor “calculation ability” was included via dummy coding. In addition, the interaction term *operationalization × calculation_ability* was modeled.

The predicted probability ${\hat{\gamma }}_{ij}$ of correctly solving a covariation task is given by:


$$\begin{array}{c} \;{\hat{\gamma }}_{ij}=\beta_{0}+\beta_{1}\;.\;operationalization_{i}+\beta_{2}\;.\;calculation\_ability_{j}\\ +\beta_{3\;}.\;operationalization_{i}\times calculation\_ability_{j}\;\;\;\;\;\left(\mathrm{model}\;2.3\right) \end{array}$$

In all models, the participant’s ID was implemented in the model as a random factor.

## 5. Results

### 5.1. Calculation

We first consider participants’ performance on conventional Bayesian reasoning tasks (i.e., calculating the positive predictive value) for both contexts and both visualization types. Table 4 shows that there obviously were no substantial differences between contexts or visualizations.

**Table 4:** Performance (standard error *SE*) on conventional Bayesian reasoning tasks (“calculation”), separated by context and visualization type.

		Visualization		ø
		Double-tree	Unit square	
Context	Mammography	45.6% (*SE* = 4.7)	47.8% (*SE* = 4.7)	46.7% (*SE* = 3.3)
	Breathalyzer	42.1% (*SE* = 4.6)	46.1% (*SE* = 4.7)	44.1% (*SE* = 3.3)
ø		43.9% (*SE* = 3.3)	47.0% (*SE* = 3.3)	45.4% (*SE* = 2.3)

It was confirmed by means of regression (model 1 above) that there were no significant differences in performance with respect to context (*ß_1_* = 0.31; *SE* = 0.39; *z* = 0.78; *p* = 0.43), or visualization (*ß_2_* = 0.35; *SE* = 0.57; *z* = 0.62; *p* = 0.53), or their interaction (*ß_3_* = −0.16; *SE* = 0.55; *z* = − 0.29; *p* = 0.77). Fixed and random effects explained *R*^2^_conditional_ = 0.70 of the variance in performance and only fixed effects explained *R*^2^_marginal_ = 0.003. Since the implemented fixed effects (visualization and context) do not explain any variance and in the absence of significant differences (RQ 1.1 and RQ 1.2), we aggregated across both factors for the following analyses of covariational skills.

### 5.2. Covariation

**Figure 5:**
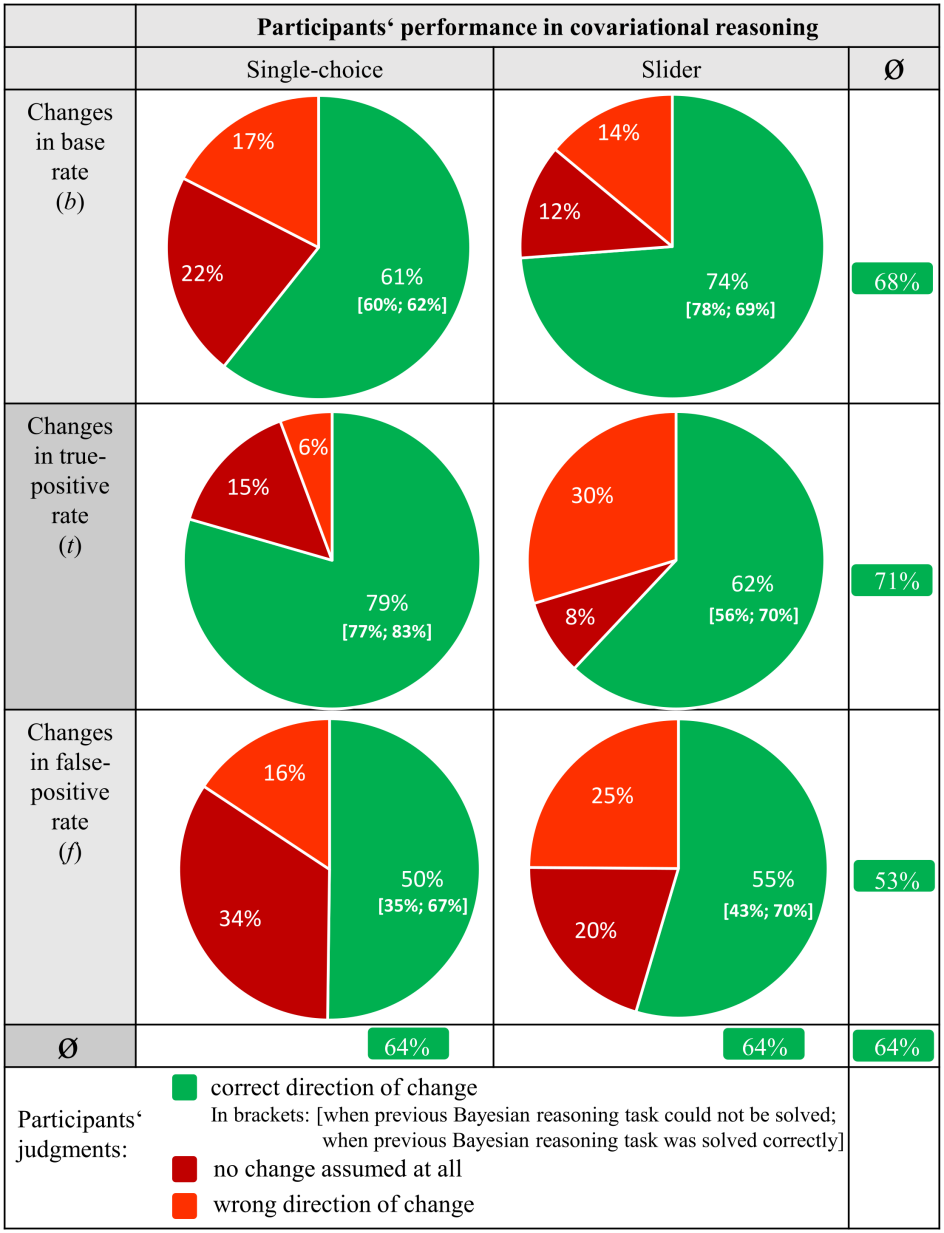
Percentages of solution rates for the three different covariation tasks (*b*, *t,* and *f*), separated by single-choice and slider.

Overall, 64% of all covariation tasks were correctly solved by participants (Figure 5) where the guessing probability was 33%. Thus, given helpful didactic tools (double-tree or unit square with frequencies), people seem in general to be capable of covariational reasoning (RQ 2.1).

While the lines in Figure 5 display performance in the three different covariation tasks (changes in *b*, *t*, and *f*), the columns distinguish the operationalizations (single-choice vs. slider). Note that we analyzed covariational reasoning across context and visualization type. However, when considering the effects of context and visualization regarding covariational reasoning, there were indeed almost no descriptive differences in solution rates across all tasks with respect to context (breathalyzer 64% vs. mammography 63%) and visualization (double-tree 63% vs. unit square 64%).

To get an initial descriptive overview of the results, some descriptive observations need to be discussed. First, estimating the effects of changes in *f* on the *PPV* (53%, see column 3, line 3) descriptively seemed to be more difficult than those of changes in the other two parameters (68% in column 3, line 1, or 71% in column 3, line 2). Second, judging the effects of changes in *b* seemed to be easier when using the slider operationalization (compare 74% vs. 61%, columns 1–2, line 1), whereas judging the effects of changes in *t* appeared to be easier when using the single-choice operationalization (62% vs. 79%, columns 1–2, line 2). Interestingly, only judging the consequences of changes in *f* did not differ substantially between the two measurement operationalizations (columns 1–2, line 3). Third, and most intriguing, an ability in conventional Bayesian reasoning (“calculation”) seemed to be most relevant concerning changes in *f* (compare Figure 5, brackets indicating: given wrong previous Bayesian reasoning; given correct previous Bayesian reasoning), which can be seen in the difference between the performances regarding covariational reasoning in the single-choice operationalization [35%; 67%] as well as in the slider operationalization [43%;70%] when evaluating changes in *f*.

Now we turn to the inferential statistics (Table 5). With respect to RQ 2.1, it can be confirmed by model 2.1 that covariational tasks regarding the judgment of effects on *PPV* were more frequently solved correctly when *b* changes than when *f* changes. Furthermore, there was no significant difference in performance between changes in *t* and changes in *b* (*R*^2^_conditional_ = 0.15, *R*^2^_marginal_ = 0.04). These results are in some ways surprising because, when *f* changes only *one* component of the visual fraction (Figure 3), changes. If *t* changes, *two* components change, and, finally, when *b* changes all *three* components change. Thus, at least from the perspective of resulting changes in the visual fraction, a change of *f* should be easiest to judge and the change of *b* most difficult.

**Table 5:** Results of the models 2.1, 2.2, and 2.3 (see 4.5).

Model			Estimate *ß*	*SE_β_*	*z*	*p*
Model 2.1 (RQ 2.1)	Comparison of evaluation of changes within the different parameters	Intercept	**0.79**	**0.11**	**6.93**	**<0.01**
		Changes in *t*	0.18	0.15	1.20	0.23
		Changes in *f*	**0.69**	**0.14**	**4.86**	**<0.001**
Model 2.2 (*b*, *t*, and *f*) (RQ 2.2)	Evaluating changes in *b* with different measurement operationalizations	Intercept	**0.48**	**0.15**	**3.15**	**<0.01**
		Operationalization	**0.66**	**0.22**	**3.06**	**<0.01**
	Evaluating changes in *t* with different measurement operationalizations	Intercept	**1.59**	**0.23**	**7.05**	**<0.001**
		Operationalization	**−1.00**	**0.24**	**−4.16**	**<0.001**
	Evaluating changes in *f* with different measurement operationalizations	Intercept	0.01	0.16	0.08	0.94
		Operationalization	0.21	0.21	1.02	0.31
Model 2.3 (RQ 2.3)	Evaluating changes in *f* with different measurement operationalizations and with no calculation ability as an additional predictor	Intercept	**−0.66**	**0.21**	**−3.13**	**<0.01**
		Operationalization	0.37	0.28	1.34	0.18
		Calculation ability	**1.41**	**0.31**	**4.55**	**<0.001**
		Operationalization × Calculation ability	−0.23	0.42	−0.55	0.58

*β* = Estimated parameter value; SE_β_ = Standard error of the parameter estimate; *z* = *z*-value; *p* = value of *p* (bold = significant at 1% level).

In order to statistically compare the effects of measurement operationalization regarding changes in *b*, *t*, or *f*, we ran three different models 2.2 (one each for a change in *b*, *t*, and *f*, respectively; Table 5). Regression analysis revealed that the type of operationalization used for measuring covariational reasoning was a significant predictor in the models of changes in *b* and *t*. For changes in *b*, the tasks with a slider operationalization were solved significantly better than the tasks with a single-choice operationalization (*R*^2^_conditional_ = 0.15, *R*^2^_marginal_ = 0.03). However, for changes in *t* (see Table 5, model 2.2), the tasks with a single-choice operationalization were solved significantly better than the tasks which were operationalized with a slider (*R*^2^_conditional_ = 0.25, *R*^2^_marginal_ = 0.06). Finally, regarding changes in *f* (see Table 5, model 2.2), the operationalization used for measuring covariational reasoning did not significantly predict the ability to answer correctly (*R*^2^_conditional_ = 0.19, *R*^2^_marginal_ = 0.003). We will return to an interpretation of these results in the discussion.

With RQ 2.3, in order to statistically estimate the effect of understanding calculation on the following covariation tasks, we ran model 2.3. The ability of conventional Bayesian reasoning indeed was a significant predictor for evaluating changes of a parameter in a Bayesian situation, but only regarding changes in *f* (Table 5; *R*^2^_conditional_ = 0.26, *R*^2^_marginal_ = 0.09). For both other changes, calculation ability was not a significant predictor (not displayed in Table 5) for the covariational reasoning. Note that only in judging changes in *f* were the directions of changes in *f* and *PPV* opposite to each other. For the other two changes (*b* and *t*), “pure intuition” seemed to suffice, even without completely understanding the situation (explanations will be provided in the discussion).

## 6. Discussion

### 6.1. Rationale and theoretical background

In the present article, we extend the research on Bayesian reasoning both theoretically and empirically with respect to the ability to deal with effects that changes in input variables (i.e., base rate *b*, true-positive rate *t*, and false-positive rate *f*) have on the positive predictive value. We chose the concept of functional thinking from mathematics education (e.g., Vollrath, 1989) as the framework for our study and theoretically explored how Bayes’ formula can be seen as a *function* (with the variables *b*, *t*, or *f*) and how the changing of parameters then refers to the *covariation* between each input variable and the *PPV*.

Measuring people’s covariational reasoning is not easy because one has to avoid just formulating a (new) conventional Bayesian reasoning task. We proposed two options to elicit covariational reasoning (i.e., the single-choice operationalization, which presented not a concrete numerical change of the input parameter but only a direction, and, alternatively, the slider operationalization, where a concrete numerical change was given but a time pressure imposed that should hinder calculating). Both operationalizations for measuring covariational reasoning were implemented in an empirical study.

In research question 1 (RQ 1), we first checked our materials to see whether the two contexts (mammography vs. breathalyzer) and/or the two visualization types (double-tree vs. unit square) would make a difference with respect to conventional Bayesian reasoning. Since we found this not to be the case, we aggregated our findings concerning covariational reasoning (RQ 2) across both contexts and both visualizations for the following analyses (when implementing contexts and visualizations in the models for RQ 2, there were no substantial differences in the statistical results, however).

### 6.2. Summary of results

First, people generally seemed to be capable of covariational reasoning when a visualization (double-tree/unit square filled with frequencies) was presented. Furthermore, estimating the effect of changes in *f* on the *PPV* (RQ 2.1) was more difficult for participants than it was with the other two parameters (*b* and *t*). This finding was surprising because, from a theoretical point of view, changes in *f* have an influence on only one component of the fraction representing the *PPV* (namely on one of the two summands in the denominator). In contrast, changes in *t* affect two elements of the fraction (numerator and one summand in the denominator) and changes in *b* all three (numerator and both summands in the denominator). It is known from the field of mathematics education that changes of independent and dependent variable in the same direction (this is the case when *b* and *t* change) are better understood than changes in the opposite direction of independent and dependet variable (this is the case when *f* changes; e.g., Hahn and Prediger, 2008). This could be a possible explanation for the surprising result. We alternatively speculate that the consequences of changes in *b* and *t* can also be grasped by intuition and without formal algorithmic reasoning.

Second, we found differences between covariational reasoning performance concerning *b* and *t* with respect to the operationalization of covariational reasoning (RQ 2.2), surprisingly, however, with a different direction of impact. While using the slider improved covariational reasoning for *b*, it made covariational reasoning for *t* worse. This might presumably be the case because, with a decrease in *t* (as in the given task), the slider had to be moved even further to the left, although the initial position in both contexts was already toward the left side of the slider (17% and 34%). Regarding the base rate change, the correct solution was to move the slider to the right (an increase in the base rate was provided in the task, and moving the slider to the right could be seen as more intuitive because of the larger space there). Comparisons between the response behavior of both operationalizations for measuring covariational reasoning could strengthen the assumption that the design of the slider operationalization impacts the response behavior. It is also noticeable that the slider was left in the original position less often compared to the option “*PPV* stays the same” in the single-choice operationalization. This could be expected, since with a slider one can ultimately choose between all values from 0% to 100%, and for that reason, one is presumably more tempted to change something. In the single-choice operationalization, in contrast, only three options were given (and thus “*PPV* stays the same” has a one-third probability of being guessed).

Third, ability in conventional Bayesian reasoning (RQ 2.3) was a predictor for covariational reasoning with respect to changes in *f* only. Regarding *b* and *t*, in contrast, people both with and without a complete understanding of conventional Bayesian reasoning can estimate the consequences (see the values in the brackets in Figure 5). This finding is in line with the results of RQ 2.1.

### 6.3. Limitations and future research

In the present study, established visualizations for calculation (double-tree and unit square) as a basis for the covariation tasks were applied in order to avoid floor effects. Nevertheless, it would be interesting to see to what extent individuals are able to solve such covariation tasks without any presentation of helpful strategies. In addition, a *systematic* comparison of different visualizations and information formats is still pending. And covariation tasks could, of course, still be considered when more than one input variable is changed. In medical tests, for example, it is typically the case that both the false-positive rate and the true-positive rate vary from one test to another. For example, if both of these probabilities increase by the same absolute percentage, one could again examine the effect on the *PPV*. In the covariation tasks that we employed, only one direction of change concerning each input variable (*b*, *t*, *f*) was implemented (in each operationalization for measuring covariational reasoning; Table 2).

Moreover, in our analyses, in order to be able to compare both operationalizations, we categorized the “participant’s variable movements” in the slider operationalization into three categories “*PPV* decreases,” “*PPV* stays the same,” and “*PPV* increases.” To judge people’s covariational reasoning skills more precisely, we might need to closely analyze how far the sliders were moved. In the same way, future research could analyze the role of the starting position of the slider.

Another problem with the slider format in our study might be that the implemented small changes in the input variables led to relatively small changes in the *PPV*. For instance, participants who thought that only a very small change was likely to happen might have decided not to move the slider at all. However, a closer look at our data revealed that when answers of “stays the same” in the slider tasks were also counted as correct, there were no significant differences in the results of all models at least with respect to changes in *b* and *t* (the results with respect to *f* are mixed). Future research could examine larger changes in terms of input variables, especially in the slider format.

Our recommendation to measure covariational reasoning in future research (especially in medical contexts, where small base rates are common) would be a combination of a single-choice task followed by justifications for the chosen direction of change. For instance, given that participants gave the correct answer in the single-choice task, they might be provided with a closed-item format with various (correct or wrong) justifications.

Regarding the results obtained, it is not clear why changes of *f* are understood worse than of *t* and *b* and why an ability in conventional Bayesian reasoning was a predictor for covariational skills only in the case of a change in *f* (although we provided speculation above). Here it would certainly be interesting to analyze the additional open justifications from our participants to capture their reasoning processes (see Büchter et al., under review). Future research might also analyze, e.g., whether such reasoning strategies depend on the concrete values of *b*, *t*, and *f* as is the case in conventional Bayesian reasoning tasks (Hafenbrädl and Hoffrage, 2015).

Furthermore, specific well-known errors in *conventional* Bayesian reasoning (see Binder et al., 2020; Eichler et al., 2020) might also explain findings regarding covariational reasoning (e.g., the high solution rates for the changes in *b* and *t*). In conventional Bayesian reasoning, for instance, the instruction for calculating the *PPV* (“to be under influence of alcohol, given a positive test result”) is sometimes misunderstood as a joint probability (“to be under the influence of alcohol *and* to get a positive test result”). If we assume that a participant wrongly thinks that the *PPV* can be described by the visual fraction denoting a joint probability, namely $\frac{\#\mathrm{true}-\mathrm{positives}\;\left(e.g.,90\right)\;}{\#\mathrm{all}\;\mathrm{persons}\;\left(e.g.,1,000\right)}$, then a change in *b* and *t* would change both the correct *PPV* and the wrong visual fraction in the same direction. Thus, participants holding this misconception would arrive at the correct answer in the covariation tasks (yielding a higher solution rate). Note that a change in *f* would have no consequences in the wrong visual fraction, and here, participants would erroneously decide that the *PPV* stays the same.

It would also be possible (and interesting) to examine covariational reasoning skills with experts in prominent applied domains such as medicine and law. Of course, in these domains a training in covariational reasoning could be constructed and implemented. In such a training on covariational reasoning, one could, for instance, work with dynamic geometry software to make changes in *b*, *t*, and *f* even more intuitive, for example by using a dynamic double-tree or a dynamic unit square (for a proposal of such dynamic visualizations see Büchter et al., 2022b; for information on a respective training course, see http://www.bayesian-reasoning.de/en/br_trainbayes_en.html or Büchter et al., 2022a).

## Data availability statement

The original contributions presented in the study are included in the article/Supplementary material, further inquiries can be directed to the corresponding author.

## Ethics statement

Ethical review and approval was not required for this study on human participants in accordance with local legislation and institutional requirements. The participants provided their written informed consent to participate in this study.

## Author contributions

Material preparation and data collection were performed by NS and TB. Data analysis was carried out by NS and KB. The first draft of the manuscript was written by NS, KB, and SK. All authors contributed to the study’s conception and design. All authors commented on previous versions of the manuscript. All authors read and approved the final manuscript.

## References

[ref1] Arkes H. R. Harkness A. R. (1983). Estimates of contingency between two dichotomous variables. J. Exp. Psychol. Gen. 112, 117–135. doi: 10.1037/0096-3445.112.1.117

[ref2] Binder K. Krauss S. Bruckmaier G. (2015). Effects of visualizing statistical information - an empirical study on tree diagrams and 2 × 2 tables. Front. Psychol. 6:1186. doi: 10.3389/fpsyg.2015.01186, PMID: 26379569 PMC4549558

[ref3] Binder K. Krauss S. Wiesner P. (2020). A new visualization for probabilistic situations containing two binary events: the frequency net. Front. Psychol. 11:750. doi: 10.3389/fpsyg.2020.00750, PMID: 32528335 PMC7264419

[ref4] Binder K. Steib N. Krauss S. (2022). Von Baumdiagrammen über Doppelbäume zu Häufigkeitsnetzen – kognitive Überlastung oder didaktische Unterstützung? [Moving from tree diagrams to double trees to net diagrams—cognitively overwhelming or educationally supportive?]. JMD, 1–33. doi: 10.1007/s13138-022-00215-9

[ref5] Böcherer-Linder K. Eichler A. (2017). The impact of visualizing nested sets. An empirical study on tree diagrams and unit squares. Front. Psychol. 7:2026. doi: 10.3389/fpsyg.2016.02026, PMID: 28123371 PMC5226638

[ref6] Böcherer-Linder K. Eichler A. (2019). How to improve performance in Bayesian inference tasks: a comparison of five visualizations. Front. Psychol. 10:267. doi: 10.3389/fpsyg.2019.00267, PMID: 30873061 PMC6401595

[ref7] Böcherer-Linder K. Eichler A. Vogel M. (2017). The impact of visualization on flexible Bayesian reasoning. AIEM 25–46. doi: 10.35763/aiem.v1i11.169

[ref8] Borovcnik M. (2012). Multiple perspectives on the concept of conditional probability. Avances de Investigación en Educación Matemática 1, 5–27. doi: 10.35763/aiem.v1i2.32

[ref9] Brase G. L. (2014). The power of representation and interpretation. Doubling statistical reasoning performance with icons and frequentist interpretations of ambiguous numbers. J. Cogn. Psychol. 26, 81–97. doi: 10.1080/20445911.2013.861840

[ref10] Brase G. L. (2021). What facilitates Bayesian reasoning? A crucial test of ecological rationality versus nested sets hypotheses. Psychon. Bull. Rev. 28, 703–709. doi: 10.3758/s13423-020-01763-2 32885405

[ref11] Büchter T. Eichler A. Böcherer-Linder K. Vogel M. Binder K. Krauss S.. (under review). Covariational reasoning in Bayesian situations. Educ. Stud. Math. 10.3389/fpsyg.2023.1184370 PMC10614641 37908812

[ref12] Büchter T. Eichler A. Steib N. Binder K. Böcherer-Linder K. Krauss S.. (2022a). How to train novices in Bayesian reasoning. Mathematics 10:1558. doi: 10.3390/math10091558

[ref13] Büchter T. Steib N. Böcherer-Linder K. Eichler A. Krauss S. Binder K.. (2022b). Designing visualizations for Bayesian problems according to multimedia principles. Education Scienes 12:739. doi: 10.3390/educsci12110739

[ref9001] Eichler A. Vogel D. (2010). Die (Bild-) Formel von Bayes [The (picture-) formula of Bayes]. PM-Praxis der Mathematik in der Schule 32:25–30.

[ref14] Eichler A. Böcherer-Linder K. Vogel M. (2020). Different visualizations cause different strategies when dealing with Bayesian situations. Front. Psychol. 11:1897. doi: 10.3389/fpsyg.2020.01897, PMID: 32973606 PMC7472875

[ref15] Garcia-Retamero R. Hoffrage U. (2013). Visual representation of statistical information improves diagnostic inferences in doctors and their patients. Soc. Sci. Med. 83, 27–33. doi: 10.1016/j.socscimed.2013.01.034, PMID: 23465201

[ref16] Gigerenzer G. Hoffrage U. (1995). How to improve Bayesian reasoning without instruction: frequency formats. Psychol. Rev. 102, 684–704. doi: 10.1037/0033-295X.102.4.684

[ref17] Hafenbrädl S. Hoffrage U. (2015). Toward an ecological analysis of Bayesian inferences: how task characteristics influence responses. Front. Psychol. 6:939. doi: 10.3389/fpsyg.2015.00939, PMID: 26300791 PMC4523724

[ref18] Hahn S. Prediger S. (2008). Bestand und Änderung — Ein Beitrag zur Didaktischen Rekonstruktion der analysis [Amount and change — a contribution to the didactic reconstruction of Calculus]. JMD 29, 163–198. doi: 10.1007/BF03339061

[ref19] Hoffrage U. Gigerenzer G. (1998). Using natural frequencies to improve diagnostic inferences. Acad. Med. 73, 538–540. doi: 10.1097/00001888-199805000-00024 9609869

[ref20] Hoffrage U. Lindsey S. Hertwig R. Gigerenzer G. (2000). Communicating statistical information. Science 290, 2261–2262. doi: 10.1126/science.290.5500.2261 11188724

[ref9005] Krauss S. Weber P. Binder K. Bruckmaier G. (2020). Natürliche Häufigkeiten als numerische Darstellungsart von Anteilen und Unsicherheit—Forschungsdesiderate und einige Antworten [Natural frequencies as a numerical representation of proportions and uncertainty—research desiderata and some answers]. Journal für Mathematik-Didaktik. 2, 485–521. doi: 10.1007/s13138-019-00156-w, PMID: 29048176

[ref21] Lindsey S. Hertwig R. Gigerenzer G. (2003). Communicating statistical DNA evidence. Jurimetrics 43, 147–163.

[ref22] McDowell M. Jacobs P. (2017). Meta-analysis of the effect of natural frequencies on Bayesian reasoning. Psychol. Bull. 143, 1273–1312. doi: 10.1037/bul0000126, PMID: 29048176

[ref23] McKenzie C. R. M. (1994). The accuracy of intuitive judgment strategies: covariation assessment and Bayesian inference. Cogn. Psychol. 26, 209–239. doi: 10.1006/cogp.1994.1007

[ref24] McKenzie C. R. M. (2004). Framing effects in inference tasks and why they are normatively defensible. Mem. Cogn. 32, 874–885. doi: 10.3758/BF03196866 15673176

[ref25] McKenzie C. R. M. Mikkelsen L. A. (2007). A Bayesian view of covariation assessment. Cogn. Psychol. 54, 33–61. doi: 10.1016/j.cogpsych.2006.04.004, PMID: 16764849

[ref26] Pfannkuch M. Budgett S. (2017). Reasoning from an Eikosogram: an exploratory study. Int. J. Res. Undergrad. Math. Ed. 3, 283–310. doi: 10.1007/s40753-016-0043-0

[ref27] Reani M. Davies A. Peek N. Jay C. (2018). How do people use information presentation to make decisions in Bayesian reasoning tasks? Int. J. Hum. Comp. Stud. 111, 62–77. doi: 10.1016/j.ijhcs.2017.11.004

[ref28] Shaklee H. Mims M. (1981). Development of rule use in judgments of covariation between events. Child Dev. 52:317. doi: 10.2307/1129245

[ref29] Sirota M. Kostovičová L. Juanchich M. (2014). The effect of iconicity of visual displays on statistical reasoning. Evidence in favor of the null hypothesis. Psychon. Bull. Rev. 21, 961–968. doi: 10.3758/s13423-013-0555-4, PMID: 24307248

[ref30] Spiegelhalter D. Pearson M. Short I. (2011). Visualizing uncertainty about the future. Science 333, 1393–1400. doi: 10.1126/science.1191181 21903802

[ref31] Talboy A. N. Schneider S. L. (2017). Improving accuracy on Bayesian inference problems using a brief tutorial. J. Behav. Dec. Making 30, 373–388. doi: 10.1002/bdm.1949

[ref32] Thompson P. W. Harel G. (2021). Ideas foundational to calculus learning and their links to students’ difficulties. ZDM 53, 507–519. doi: 10.1007/s11858-021-01270-1

[ref33] Vollrath H.-J. (1989). Funktionales Denken [Functional thinking]. JMD 10, 3–37. doi: 10.1007/bf03338719

[ref34] Zhu L. Gigerenzer G. (2006). Children can solve Bayesian problems: the role of representation in mental computation. Cognition 98, 287–308. doi: 10.1016/j.cognition.2004.12.003, PMID: 16399266

