# Supplementary material for: Measuring people’s covariational reasoning in Bayesian situations
Source: Front Psychol. 2023 Oct 16;14:1184370. doi: 10.3389/fpsyg.2023.1184370 (PMC10614641; doi:10.3389/fpsyg.2023.1184370)
Supplement: Supplementary file 1 [file Data_Sheet_1.PDF]

Introductory Example for the double-tree

This study is about the double-tree. The aim of this introductory example is to learn how to read out information from a double-tree. Please read this introductory example carefully and answer the questions afterwards. Thank you for your cooperation.

Introductory example:

In a class of pupils boys and girls are asked whether or not they play football. The results are presented in a tree-diagram:

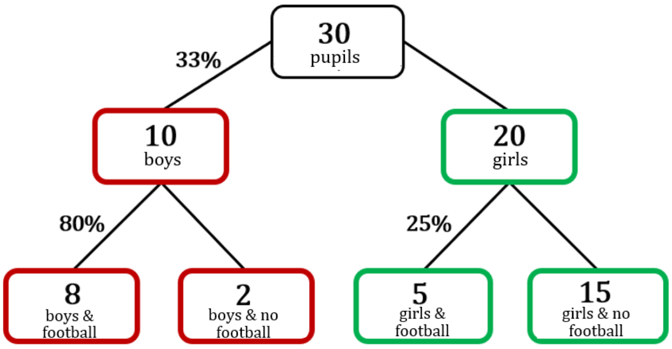

With the so-called double-tree, you can simultaneously visualize the division of the sample into "football" and "no football". For that, all boys and girls who play football are grouped together on the left (13 pupils in total) and all boys and girls who do not play football are grouped together on the right (17 pupils in total):

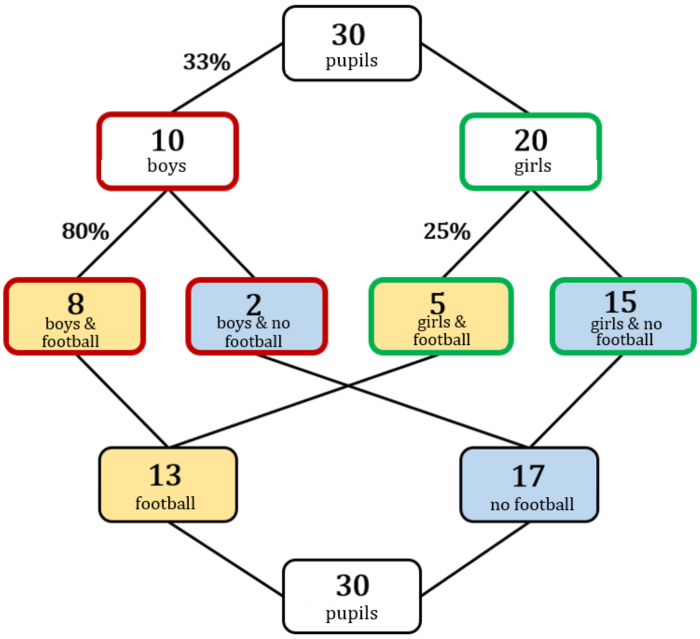

Now, you are familiar with the double-tree and are ready to work on the questions. You can download this introductory example here. Later, you cannot access this example anymore.

Did you already know the visualization which was presented here?

□ Yes □ No
