# Supplementary material for: Measuring people’s covariational reasoning in Bayesian situations
Source: Front Psychol. 2023 Oct 16;14:1184370. doi: 10.3389/fpsyg.2023.1184370 (PMC10614641; doi:10.3389/fpsyg.2023.1184370)
Supplement: Supplementary file 2 [file Data_Sheet_2.PDF]

Introductory Example for the unit square

This study is about the unit square. The aim of this introductory example is to learn how to read out information from a unit square. Please read this introductory example carefully and answer the questions afterwards. Thank you for your cooperation.

Introductory example:

In a class of pupils, boys and girls are asked whether or not they play football. Results:

	Boys	Girls	Sum
Football	8	5	13
No football	2	15	17
Sum	10	20	30

With the unit square you can visualize this information in such a way that the size of the inner areas corresponds to the proportion within the areas. For that, the square is first divided vertically into the proportion of boys and girls respectively:

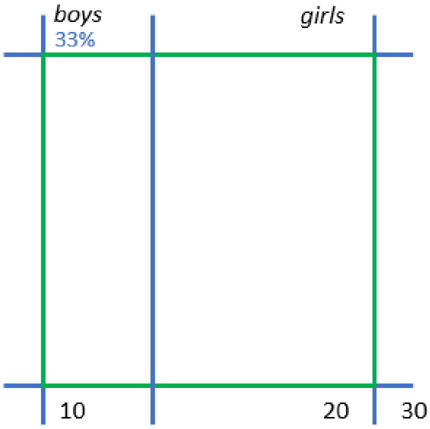

Afterwards, the two areas for boys and girls are again divided into the ones who play „football" or play „no football" according to the ratio of the corresponding frequencies:

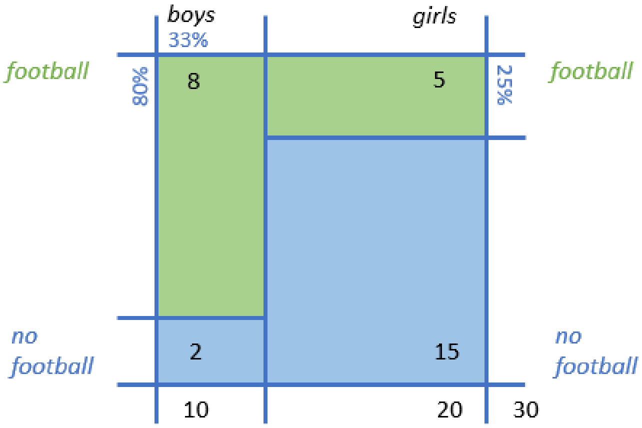

Now, you are familiar with the unit square and are ready to work on the questions. You can download this introductory example here. Later, you cannot access this example anymore.

Did you already know the visualization which was presented here?

☐ Yes ☐ No
