# Supplementary material for: Measuring people’s covariational reasoning in Bayesian situations
Source: Front Psychol. 2023 Oct 16;14:1184370. doi: 10.3389/fpsyg.2023.1184370 (PMC10614641; doi:10.3389/fpsyg.2023.1184370)
Supplement: Supplementary file 3 [file Data_Sheet_3.pdf]

Mammography Screening

In Hessen every year, about 1,000 women who have no symptoms of breast cancer and who have no near relatives known to have had breast cancer participate in a mammography screening. Of these women only a small proportion actually has breast cancer. In the mammography screenings a large proportion of the women with breast cancer is detected and therefore tests positive. A small proportion of the women without breast cancer is falsely tested positive.

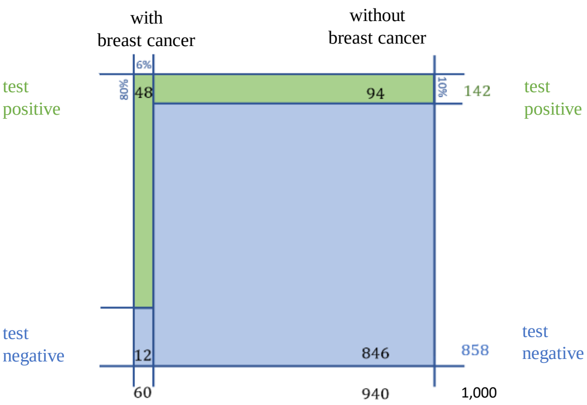

How likely has a woman actually breast cancer, if she tests positive in the mammography screening?

In order to calculate this probability, you have to form a fraction (numerator/denominator). Please determine:

Numerator (as a whole number): ______

Denominator (as a whole number): ______

Probability (in percent with 2 decimals): ______ %

In the following three tasks, you are asked to consider how changes in statistical information affect the situation.
The tasks each relate to the situation above (values in the visualization). You can see in each subtask the visualization above and the question.

Imagine, the probability that a woman has breast cancer is smaller than 6%. The other values are the same as in the visualization.

How does that change the probability that a woman actually has breast cancer, if she tests positive in the mammography screening (compared to the original situation in the visualization)?

The probability … decreases ○ stays the same ○ increases ○

Imagine, the probability that a woman with breast cancer tests positive is larger than 80%. The other values are the same as in the visualization.

How does that change the probability that a woman actually has breast cancer, if she tests positive in the mammography screening (compared to the original situation in the visualization)?

The probability … decreases ○ stays the same ○ increases ○

Imagine, the probability that a woman without breast cancer falsely tests positive is larger than 10%. The other values are the same as in the visualization.

How does that change the probability that a woman actually has breast cancer, if she tests positive in the mammography screening (compared to the original situation in the visualization)?

The probability … decreases ○ stays the same ○ increases ○

All three tasks were completed in random order by the particpants.

Breathalyzer tests

In traffic stops last August in Regensburg, 1,000 drivers were tested with a breathalyzer test for checking their intoxication levels. In Regensburg, only a small proportion of the drivers is under the influence of alcohol. The test Dräger-6510 has the following characteristics: the majority of the people, who are under influence of alcohol, is detected with the breathalyzer test and therefore tests positive. A large proportion of the people, who are not under the influence of alcohol, test positive nevertheless.

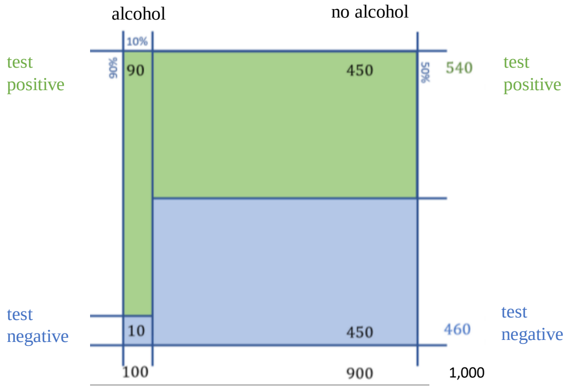

How likely is a person actually under the influence of alcohol, if he or she tests positive in the breathalyzer test?

In order to calculate this probability, you have to form a fraction (numerator/denominator). Please determine:

Numerator (as a whole number): ______

Denominator (as a whole number): ______

Probability (in percent with 2 decimals): ______ %

In the following three tasks, you are asked to consider how changes in statistical information affect the situation.
The tasks each relate to the situation above (values in the visualization). You can see in each subtask the visualization above and the question.

All three tasks were completed in random order by the particpants.

Imagine, the probability that a person is under the influence of alcohol is smaller than 10%. The other values are the same as in the visualization.

How does that change the probability that a person is actually under the influence of alcohol, if he or she tests positive in the breathalyzer test (compared to the original situation in the visualization)?

The probability … decreases ◯ stays the same ◯ increases ◯

Imagine, the probability that a person, who is under the influence of alcohol, tests positive is larger than 90%. The other values are the same as in the visualization.

How does that change the probability that a person is actually under the influence of alcohol, if he or she tests positive in the breathalyzer test (compared to the original situation in the visualization)?

The probability … decreases ◯ stays the same ◯ increases ◯

Imagine, the probability that a person, who is not under the influence of alcohol, falsely tests positive is larger than 50%. The other values are the same as in the visualization.

How does that change the probability that a person is actually under the influence of alcohol, if he or she tests positive in the breathalyzer test (compared to the original situation in the visualization)?

The probability … decreases ◯ stays the same ◯ increases ◯

Mammography Screening

In Hessen every year, about 1,000 women who have no symptoms of breast cancer and who have no near relatives known to have had breast cancer participate in a mammography screening. Of these women only a small proportion actually has breast cancer. In the mammography screenings a large proportion of the women with breast cancer is detected and therefore tests positive. A small proportion of the women without breast cancer is falsely tested positive.

How likely has a woman actually breast cancer, if she tests positive in the mammography screening?

In order to calculate this probability, you have to form a fraction (numerator/denominator). Please determine:

Numerator (as a whole number): ______

Denominator (as a whole number): ______

Probability (in percent with 2 decimals): ______ %

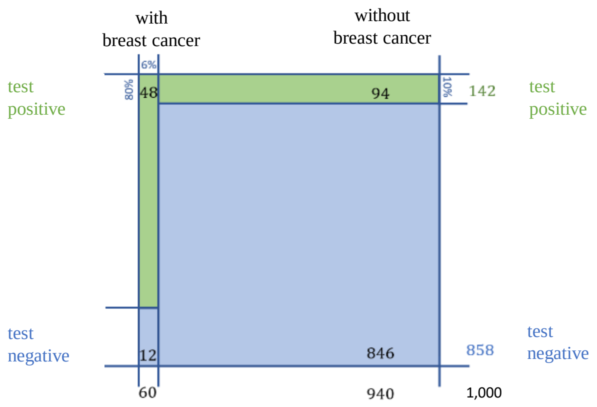

In the following three tasks, you are asked to consider how changes in statistical information affect the situation. The tasks each relate to the situation above (values in the visualization). You can see the visualization above and the question in each subtask. You are to complete the next three questions as quickly as possible.

All three tasks were completed in random order by the particpants.

Imagine, the probability that a woman has breast cancer is 2% larger than 6%. The other values are the same as in the visualization.

What do you estimate: How likely has a women then actually breast cancer, if she tests positive in the mammography screening (compared to the original situation in the visualization)?

Reply as quickly as possible by moving the slider.

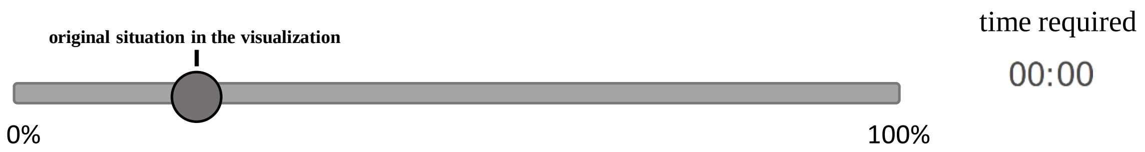

If you do not move the slider, the probability stays the same.

Imagine, the probability that a woman with breast cancer tests positive is 3% smaller than 80%. The other values are the same as in the visualization.

What do you estimate: How likely has a women then actually breast cancer, if she tests positive in the mammography screening (compared to the original situation in the visualization)?

Reply as quickly as possible by moving the slider.

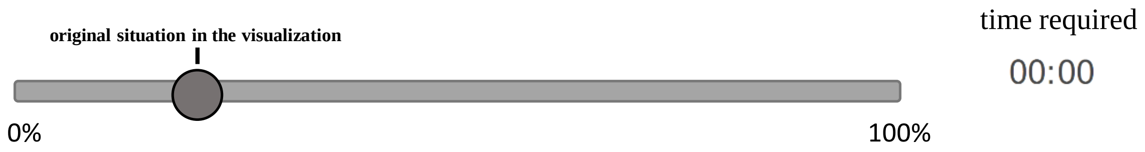

If you do not move the slider, the probability stays the same.

Imagine, the probability that a woman without breast cancer falsely tests positive is 3% smaller than 10%. The other values are the same as in the visualization.

What do you estimate: How likely has a women then actually breast cancer, if she tests positive in the mammography screening (compared to the original situation in the visualization)?

Reply as quickly as possible by moving the slider.

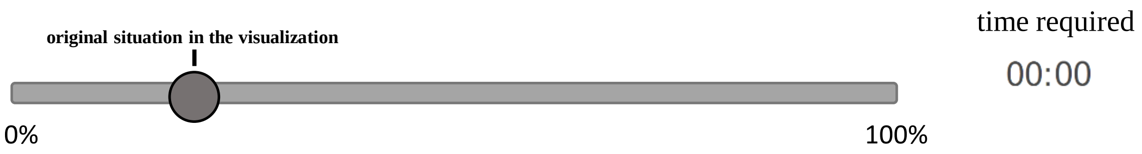

If you do not move the slider, the probability stays the same.

Breathalyzer tests

In traffic stops last August in Regensburg, 1,000 drivers were tested with a breathalyzer test for checking their intoxication levels. In Regensburg, only a small proportion of the drivers is under the influence of alcohol. The test Dräger-6510 has the following characteristics: the majority of the people, who are under influence of alcohol, is detected with the breathalyzer test and therefore tests positive. A large proportion of the people, who are not under the influence of alcohol, test positive nevertheless.

How likely is a person actually under the influence of alcohol, if he or she tests positive in the breathalyzer test?

In order to calculate this probability, you have to form a fraction (numerator/denominator). Please determine:

Numerator (as a whole number):

Denominator (as a whole number):

Probability (in percent with 2 decimals): %

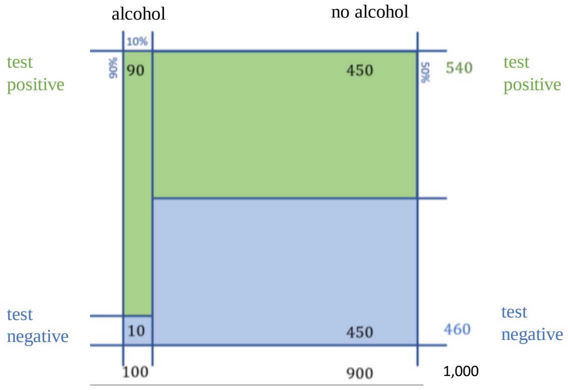

In the following three tasks, you are asked to consider how changes in statistical information affect the situation. The tasks each relate to the situation above (values in the visualization). You can see the visualization above and the question in each subtask. You are to complete the next three questions as quickly as possible.

All three tasks were completed in random order by the particpants.

Imagine, the probability that a person is under the influence of alcohol is 2% larger than 10%. The other values are the same as in the visualization.

What do you estimate: How likely is a person then actually under the influence of alcohol, if he or she tests positive in the breathalyzer test (compared to the original situation in the visualization)?

Reply as quickly as possible by moving the slider.

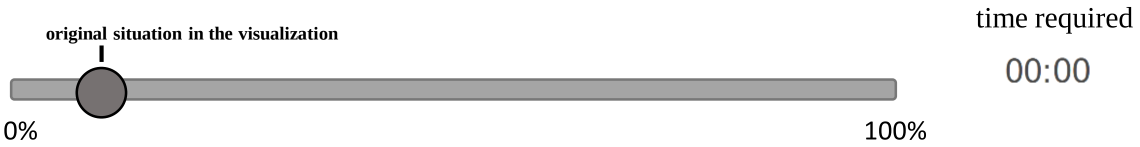

If you do not move the slider, the probability stays the same.

Imagine, the probability that a person, who is under the influence of alcohol, tests positive is 3% smaller than 90%. The other values are the same as in the visualization.

What do you estimate: How likely is a person then actually under the influence of alcohol, if he or she tests positive in the breathalyzer test (compared to the original situation in the visualization)?

Reply as quickly as possible by moving the slider.

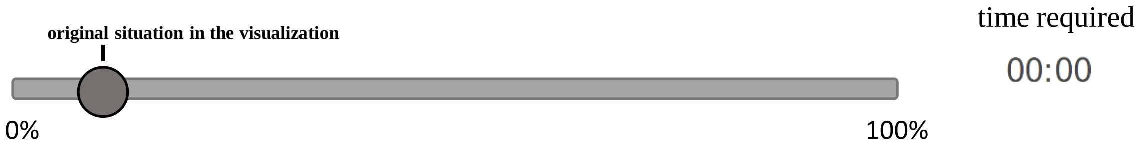

If you do not move the slider, the probability stays the same.

Imagine, the probability that a person, who is not under the influence of alcohol, falsely tests positive is 3% smaller than 50%. The other values are the same as in the visualization.

What do you estimate: How likely is a person then actually under the influence of alcohol, if he or she tests positive in the breathalyzer test (compared to the original situation in the visualization)?

Reply as quickly as possible by moving the slider.

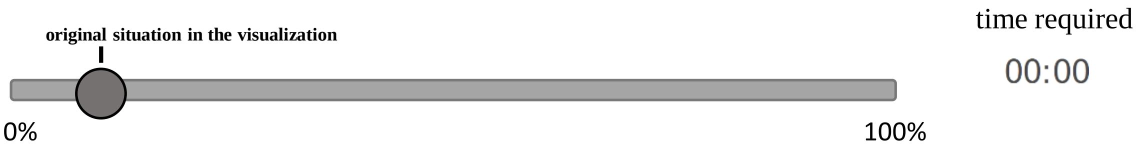

If you do not move the slider, the probability stays the same.

Mammography Screening

In Hessen every year, about 1,000 women who have no symptoms of breast cancer and who have no near relatives known to have had breast cancer participate in a mammography screening. Of these women only a small proportion actually has breast cancer. In the mammography screenings a large proportion of the women with breast cancer is detected and therefore tests positive. A small proportion of the women without breast cancer is falsely tested positive.

How likely has a woman actually breast cancer, if she tests positive in the mammography screening?

In order to calculate this probability, you have to form a fraction (numerator/denominator). Please determine:

Numerator (as a whole number):

Denominator (as a whole number):

Probability (in percent with 2 decimals): %

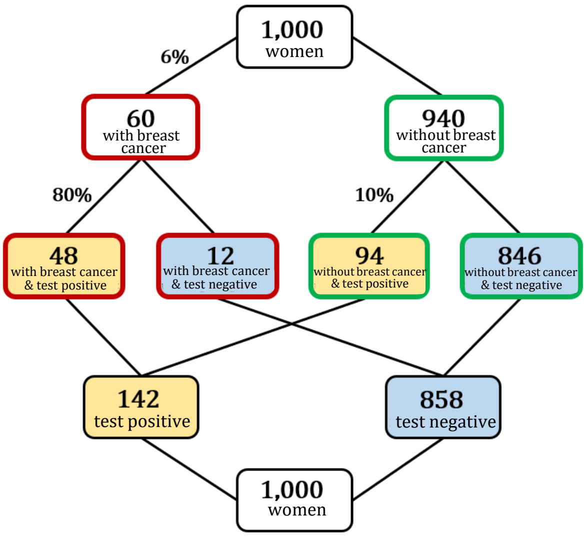

In the following three tasks, you are asked to consider how changes in statistical information affect the situation.
The tasks each relate to the situation above (values in the visualization). You can see in each subtask the visualization above and the question.

All three tasks were completed in random order by the particpants.

Imagine, the probability that a woman has breast cancer is smaller than 6%. The other values are the same as in the visualization.

How does that change the probability that a woman actually has breast cancer, if she tests positive in the mammography screening (compared to the original situation in the visualization)?

The probability … decreases ○ stays the same ○ increases ○

Imagine, the probability that a woman with breast cancer tests positive is larger than 80%. The other values are the same as in the visualization.

How does that change the probability that a woman actually has breast cancer, if she tests positive in the mammography screening (compared to the original situation in the visualization)?

The probability … decreases ○ stays the same ○ increases ○

Imagine, the probability that a woman without breast cancer falsely tests positive is larger than 10%. The other values are the same as in the visualization.

How does that change the probability that a woman actually has breast cancer, if she tests positive in the mammography screening (compared to the original situation in the visualization)?

The probability … decreases ○ stays the same ○ increases ○

Breathalyzer tests

In traffic stops last August in Regensburg, 1,000 drivers were tested with a breathalyzer test for checking their intoxication levels. In Regensburg, only a small proportion of the drivers is under the influence of alcohol. The test Dräger-6510 has the following characteristics: the majority of the people, who are under influence of alcohol, is detected with the breathalyzer test and therefore tests positive. A large proportion of the people, who are not under the influence of alcohol, test positive nevertheless.

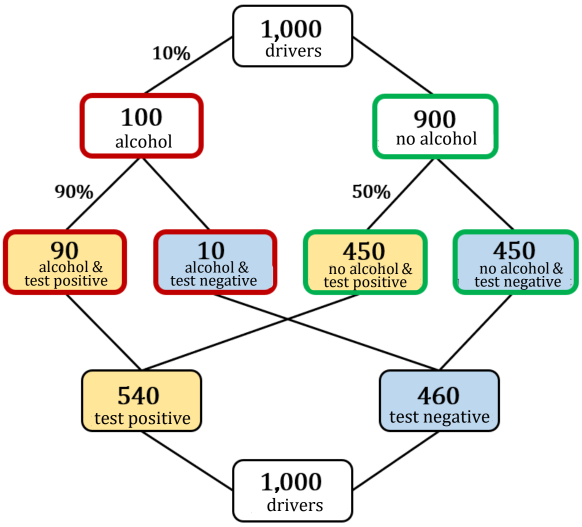

How likely is a person actually under the influence of alcohol, if he or she tests positive in the breathalyzer test?

In order to calculate this probability, you have to form a fraction (numerator/denominator). Please determine:

Numerator (as a whole number):

Denominator (as a whole number):

Probability (in percent with 2 decimals): %

In the following three tasks, you are asked to consider how changes in statistical information affect the situation.
The tasks each relate to the situation above (values in the visualization). You can see in each subtask the visualization above and the question.

All three tasks were completed in random order by the particpants.

Imagine, the probability that a person is under the influence of alcohol is smaller than 10%. The other values are the same as in the visualization.

How does that change the probability that a person is actually under the influence of alcohol, if he or she tests positive in the breathalyzer test (compared to the original situation in the visualization)?

The probability … decreases ○ stays the same ○ increases ○

Imagine, the probability that a person, who is under the influence of alcohol, tests positive is larger than 90%. The other values are the same as in the visualization.

How does that change the probability that a person is actually under the influence of alcohol, if he or she tests positive in the breathalyzer test (compared to the original situation in the visualization)?

The probability … decreases ○ stays the same ○ increases ○

Imagine, the probability that a person, who is not under the influence of alcohol, falsely tests positive is larger than 50%. The other values are the same as in the visualization.

How does that change the probability that a person is actually under the influence of alcohol, if he or she tests positive in the breathalyzer test (compared to the original situation in the visualization)?

The probability … decreases ○ stays the same ○ increases ○

Mammography Screening

In Hessen every year, about 1,000 women who have no symptoms of breast cancer and who have no near relatives known to have had breast cancer participate in a mammography screening. Of these women only a small proportion actually has breast cancer. In the mammography screenings a large proportion of the women with breast cancer is detected and therefore tests positive. A small proportion of the women without breast cancer is falsely tested positive.

How likely has a woman actually breast cancer, if she tests positive in the mammography screening?

In order to calculate this probability, you have to form a fraction (numerator/denominator). Please determine:

Numerator (as a whole number): ______

Denominator (as a whole number): ______

Probability (in percent with 2 decimals): ______ %

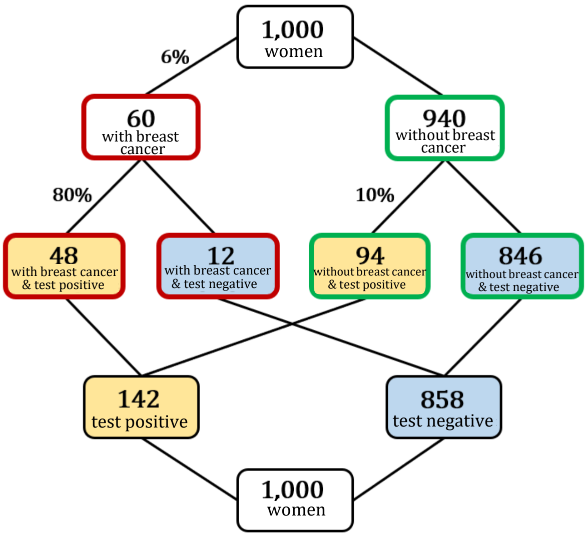

In the following three tasks, you are asked to consider how changes in statistical information affect the situation. The tasks each relate to the situation above (values in the visualization). You can see the visualization above and the question in each subtask. You are to complete the next three questions as quickly as possible.

All three tasks were completed in random order by the particpants.

Imagine, the probability that a woman has breast cancer is 2% larger than 6%. The other values are the same as in the visualization.

What do you estimate: How likely has a women then actually breast cancer, if she tests positive in the mammography screening (compared to the original situation in the visualization)?

Reply as quickly as possible by moving the slider.

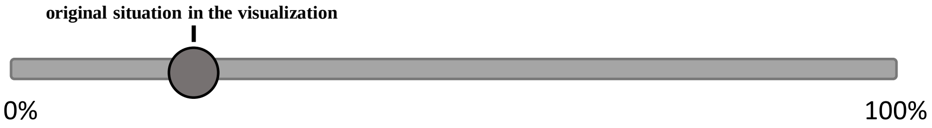

time required

00:00

If you do not move the slider, the probability stays the same.

Imagine, the probability that a woman with breast cancer tests positive is 3% smaller than 80%. The other values are the same as in the visualization.

What do you estimate: How likely has a women then actually breast cancer, if she tests positive in the mammography screening (compared to the original situation in the visualization)?

Reply as quickly as possible by moving the slider.

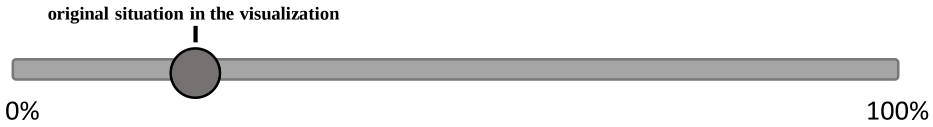

time required

00:00

If you do not move the slider, the probability stays the same.

Imagine, the probability that a woman without breast cancer falsely tests positive is 3% smaller than 10%. The other values are the same as in the visualization.

What do you estimate: How likely has a women then actually breast cancer, if she tests positive in the mammography screening (compared to the original situation in the visualization)?

Reply as quickly as possible by moving the slider.

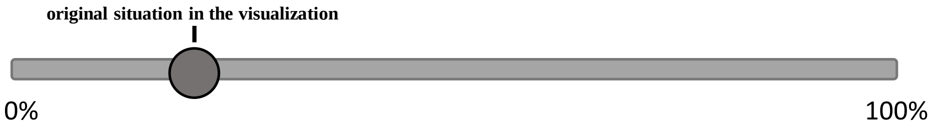

time required

00:00

If you do not move the slider, the probability stays the same.

Breathalyzer tests

In traffic stops last August in Regensburg, 1,000 drivers were tested with a breathalyzer test for checking their intoxication levels. In Regensburg, only a small proportion of the drivers is under the influence of alcohol. The test Dräger-6510 has the following characteristics: the majority of the people, who are under influence of alcohol, is detected with the breathalyzer test and therefore tests positive. A large proportion of the people, who are not under the influence of alcohol, test positive nevertheless.

How likely is a person actually under the influence of alcohol, if he or she tests positive in the breathalyzer test?

In order to calculate this probability, you have to form a fraction (numerator/denominator). Please determine:

Numerator (as a whole number): ______

Denominator (as a whole number): ______

Probability (in percent with 2 decimals): ______ %

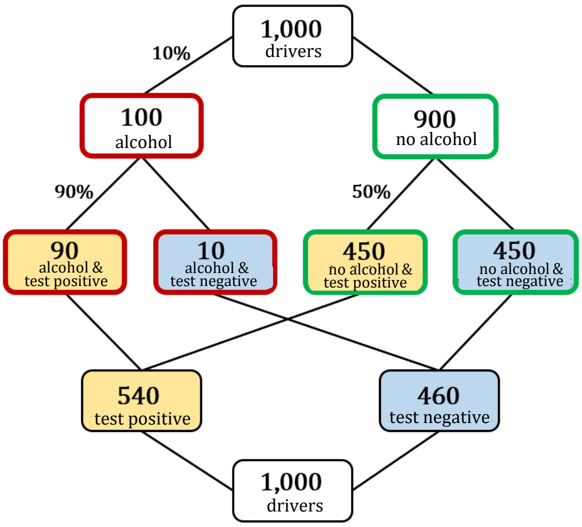

In the following three tasks, you are asked to consider how changes in statistical information affect the situation. The tasks each relate to the situation above (values in the visualization). You can see the visualization above and the question in each subtask. You are to complete the next three questions as quickly as possible.

All three tasks were completed in random order by the particpants.

Imagine, the probability that a person is under the influence of alcohol is 2% larger than 10%. The other values are the same as in the visualization.

What do you estimate: How likely is a person then actually under the influence of alcohol, if he or she tests positive in the breathalyzer test (compared to the original situation in the visualization)?

Reply as quickly as possible by moving the slider.

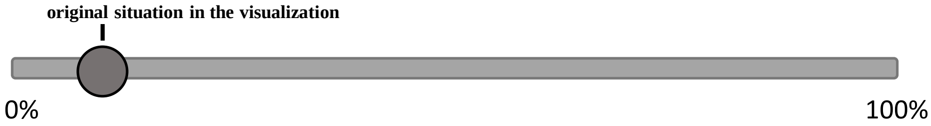

time required

00:00

If you do not move the slider, the probability stays the same.

Imagine, the probability that a person, who is under the influence of alcohol, tests positive is 3% smaller than 90%. The other values are the same as in the visualization.

What do you estimate: How likely is a person then actually under the influence of alcohol, if he or she tests positive in the breathalyzer test (compared to the original situation in the visualization)?

Reply as quickly as possible by moving the slider.

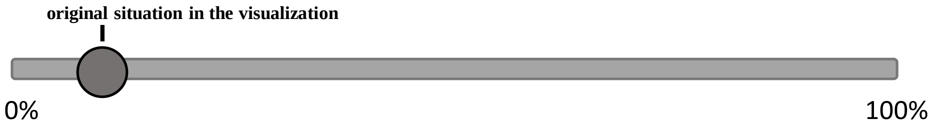

time required

00:00

If you do not move the slider, the probability stays the same.

Imagine, the probability that a person, who is not under the influence of alcohol, falsely tests positive is 3% smaller than 50%. The other values are the same as in the visualization.

What do you estimate: How likely is a person then actually under the influence of alcohol, if he or she tests positive in the breathalyzer test (compared to the original situation in the visualization)?

Reply as quickly as possible by moving the slider.

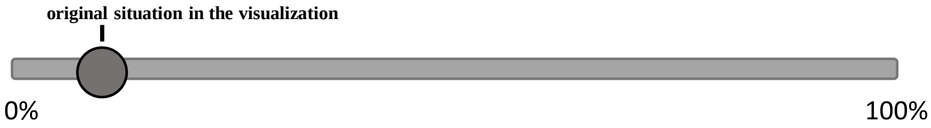

time required

00:00

If you do not move the slider, the probability stays the same.
